# ABCG2/BCRP transport mechanism revealed through kinetically excited targeted molecular dynamics simulations

**DOI:** 10.1016/j.csbj.2022.07.035

**Published:** 2022-07-29

**Authors:** B. Dudas, X. Decleves, S. Cisternino, D. Perahia, M.A. Miteva

**Affiliations:** a Inserm U1268 MCTR, CiTCoM UMR 8038 CNRS, Université Paris Cité, Paris, France; b Laboratoire de Biologie et Pharmacologie Appliquée, Ecole Normale Supérieure Paris-Saclay, Gif-sur-Yvette, France; c Inserm UMRS 1144, Optimisation Thérapeutique en Neuropsychopharmacologie - Université Paris Cité, Paris, France; d Biologie du Médicament et Toxicologie, Assistance Publique Hôpitaux de Paris, AP-HP, Hôpital Universitaire Cochin, Paris, France; e Service Pharmacie, Assistance Publique Hôpitaux de Paris, AP-HP, Hôpital Universitaire Necker-Enfants Malades, Paris, France

**Keywords:** ABC transporters, BCRP, ABCG2, Efflux mechanism, Molecular dynamics simulations, Drug-drug interactions

## Abstract

ABCG2/BCRP is an ABC transporter that plays an important role in tissue protection by exporting endogenous substrates and xenobiotics. ABCG2 is of major interest due to its involvement in multidrug resistance (MDR), and understanding its complex efflux mechanism is essential to preventing MDR and drug-drug interactions (DDI). ABCG2 export is characterized by two major conformational transitions between inward- and outward-facing states, the structures of which have been resolved. Yet, the entire transport cycle has not been characterized to date. Our study bridges the gap between the two extreme conformations by studying connecting pathways. We developed an innovative approach to enhance molecular dynamics simulations, ‘kinetically excited targeted molecular dynamics’, and successfully simulated the transitions between inward- and outward-facing states in both directions and the transport of the endogenous substrate estrone 3-sulfate. We discovered an additional pocket between the two substrate-binding cavities and found that the presence of the substrate in the first cavity is essential to couple the movements between the nucleotide-binding and transmembrane domains. Our study shed new light on the complex efflux mechanism, and we provided transition pathways that can help to identify novel substrates and inhibitors of ABCG2 and probe new drug candidates for MDR and DDI.

## Graphical abstract

**Figure:**
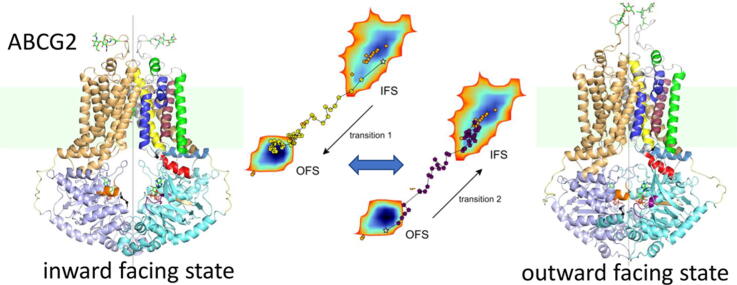

## 1 Introduction

ATP-binding cassette (ABC) transporters are molecular machineries that harvest energy from ATP hydrolysis to translocate substrates across membranes selectively [1]. Some members of the ABCB, ABCC, and ABCG subfamilies are involved in drug transport and are responsible for unidirectional drug efflux. They are of major interest due to their involvement in the multidrug resistance (MDR) phenotype of tumor cells as well as the controlling of drug pharmacokinetics at several critical body interfaces, considering their physiological expression in cells like endothelial brain cells and enterocytes [2], [3]. Furthermore, inhibition of ABC transporters and drug metabolizing enzymes [4], [5], [6] can lead to drug-drug interactions (DDI) and influence drug efficacy and safety [7].

Human ABCG2, also known as BCRP (Breast Cancer Resistance Protein), belongs to the G-subfamily of ABC transporters and is physiologically expressed in tissue barriers like the blood–brain barrier [1], [2], [8], [9], [10]. It plays an important role in tissue protection by selectively exporting numerous endogenous substrates and a broad variety of xenobiotics to extracellular spaces like the blood lumen at the blood–brain barrier [11], [12], [13]. Similar to P–glycoprotein (ABCB1) and MRPs (ABCCs), ABCG2 has also been identified as a contributor to MDR in tumor cells [14], [15], [16], [17]. ABCG2 can strongly influence the pharmacokinetic profile of a wide range of drugs due to its substrate poly-specificity. Interestingly, ABCG2 substrates comprise a broad spectrum of anticancer agents, sulfate and glucuronide conjugates of sterols and drugs that are common products of mammalian Phase II metabolism [17]. Therefore, drug agencies worldwide (e.g. the European Medicines Agency and the United States Food and Drug Administration) recommended testing for possible ABCG2 substrate or inhibitor status over the course of drug development [18], [19], [20]. It is crucial to understand the molecular mechanism of the underlying ABCG2 substrate export in all its complexity to better predict and prevent ABCG2-involved drug pharmacokinetic variability.

**Fig. 1:**
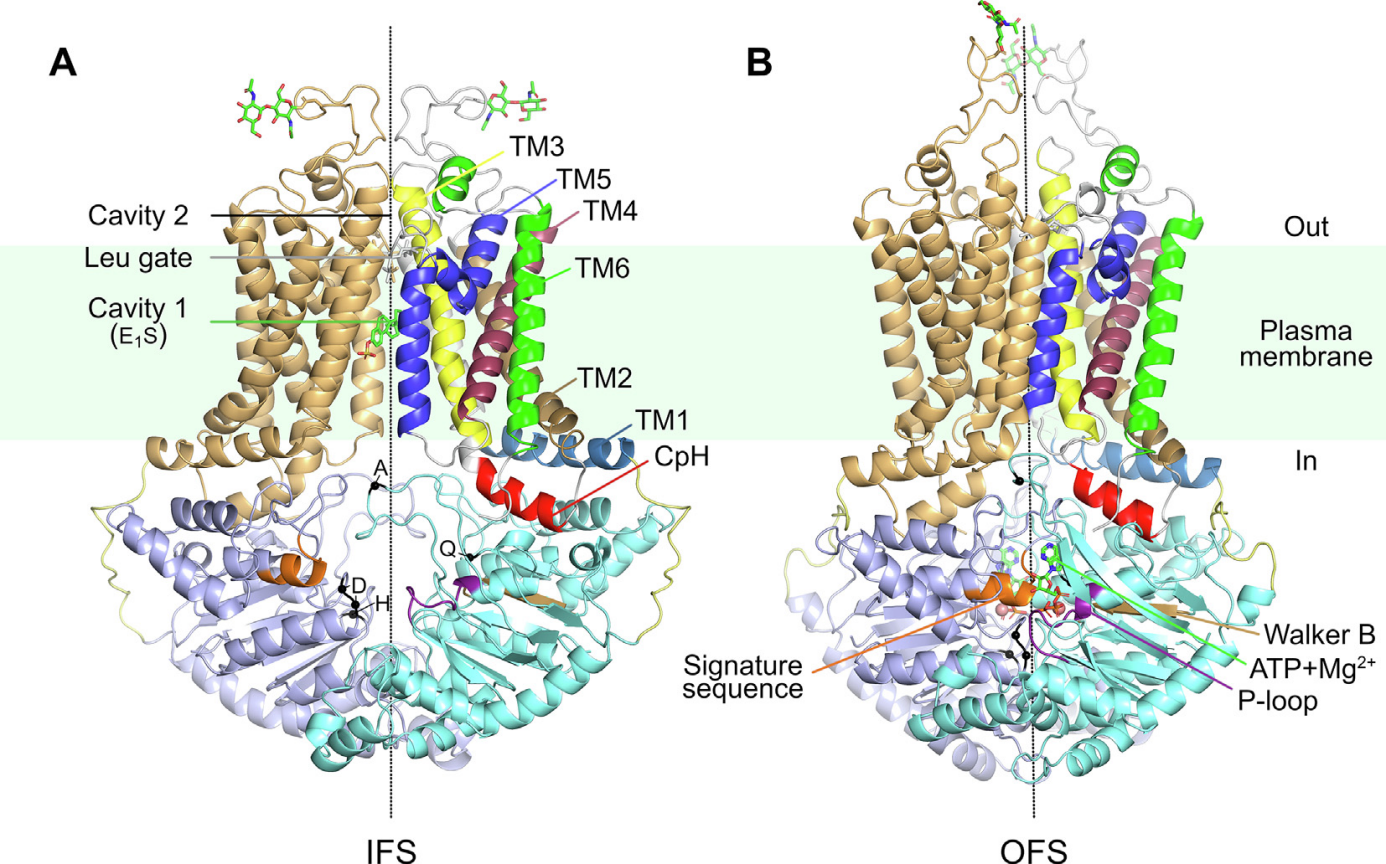
Experimental ABCG2 structure in (A) the E_1_S substrate-bound IFS (PDB 6HCO) and (B) the ATP-Mg^2+^-bound OFS (PDB 6HBU). The loop regions modelled here are shown for clarity. The rotational symmetry axis of the homodimer is indicated by a dashed line. Each monomer consists of a TMD and an NBD (e.g. TMD in light orange and NBD in light blue of one monomer). Conserved motifs within the NBDs are marked with letters (A-loop, Q-loop, d-loop, and H-loop). The “coupling helix” (CpH) of one monomer is highlighted in red, the different TM helices are highlighted in different colors. The linker segments connecting the individual NBDs and TMDs are in pale yellow. The ATPs, the substrate, the leucine gate, and the glycosyl groups are in licorice, the Mg^2+^ ions in sphere representations. Signature sequence, P-loop, and Walker B motif are also colored orange, purple, and tan, respectively. (For interpretation of the references to color in this figure legend, the reader is referred to the web version of this article.)

Conformational changes are driving forces for the substrate efflux in ABC transporters [21], [22], [23]. Over recent years, thanks to breakthrough advances in single-particle cryogenic electron microscopy (cryo-EM), several transporter structures have been resolved at a nearly atomic resolution under different conditions [21], [24], [25], [26], [27], [28]. These recent studies have identified two distinct conformational clusters of ABCG2, the transporter in the inward facing state (IFS) and the outward facing state (OFS). During the transport cycle, ABCG2 is thought to cycle between these two states [26]. ABCG2 functions as a homodimer, with each monomer consisting of a nucleotide–binding domain (NBD) and an integral transmembrane domain (TMD) (Fig. 1). NBDs contain highly conserved motifs shared among ABC transporters and can bind two ATP molecules and coordinating Mg^2+^ ions at their dimer interface. TMDs are involved in substrate recognition by forming two substrate-binding cavities (Fig. 1A). Substrates have access to cavity 1 from both the cytosol and the lipid bilayer. As opposed to cavity 1, cavity 2 faces the extracellular space, and the two cavities are separated by the so-called leucine gate (also referred to as the leucine plug) [21].

To date, neither experimental structures with a substrate bound to cavity 2, nor transient structures along the translocation pathway and the transport cycle have been resolved. Therefore, the transporter’s dynamics, playing a key role in the complex mechanism of drug efflux, needs to be elucidated. *In silico* approaches, in particular Molecular Dynamics (MD) simulations, are powerful tools in the exploration of related mechanisms [29], [30], [31], [32]. Yet, classical MD simulations fall short of providing a full atomic description of cooperative events at time scales beyond microseconds, let alone the timeframe of the transport cycle, for multi-domain systems. Although Nagy et al. investigated key interactions along the uric acid substrate-translocation pathway and its regulation by cholesterol with the help of metadynamics simulations [33], the entire ABCG2 transport cycle has not been thoroughly understood, and the dynamical behavior of the different transport stages has not been characterized to date.

To better understand the molecular mechanism of substrate export in all its complexity, here we explore the ABCG2 transition pathways of the transport cycle. Our study bridges the gap between the different transport states by employing an innovative simulation approach, starting from available experimental structures. We developed an enhanced MD simulation methodology to trace possible pathways between two terminal structures, termed ‘kinetically excited targeted Molecular Dynamics’, and successfully simulated transitions between the IFS and the OFS in both directions, along with the translocation of the physiological estrone 3-sulfate (E_1_S) substrate. Furthermore, we characterized the dynamical behavior of ABCG2 in the different transport stages.

## 2 Results and discussion

### 2.1 Structural models and kinetically excited targeted MD

We performed simulations starting from cryo-EM structures [21] of the human homodimer of ABCG2 in its IFS and OFS (see Materials and Methods for details). The structure of ABCG2 contains highly conserved motifs shared among ABC transporters at their NBDs, such as the P-loop (Walker A motif), the Walker B motif, the signature sequence (‘VSGGERKR’), and the A- and H-loops primarily responsible for ATP binding and hydrolysis, as well as the Q- and the d-loops responsible for NBD dimer formation or interdomain communication [34]. Two ATP molecules and coordinating Mg^2+^ ions have been found to bind symmetrically at the catalytic interface formed by the two NBDs, each between the P-loop of one monomer and the signature sequence of the other [21]. In the IFS, the two NBD monomers are partially separated, yet some contacts are maintained at the cytosolic tip of the transporter. The degree of NBD separation varies between the available experimental structures, from fully-inward open (e.g. nucleotide-free E_1_S-bound transporter [21]) to more, but not completely closed states (e.g. E_1_S- or topotecan-bound transporter in the presence of ATP [28]). The TMD pair forms the slit-like hydrophobic cavity 1, where the physiological E_1_S substrate and various inhibitors have been proven to bind [21], [25], [26], [27], [28]. In contrast, in the OFS, cavity 1 is completely collapsed, and the NBDs form a tightly closed interface (Fig. 1B).

We chose to model the unresolved flexible intracellular loop regions in the NBDs and include them in our simulations since they are likely to affect the substrate entry and may possess a similar gating function to analogous regions in bacterial transporters [35], [36], [37] (e.g. the loop region between the first and second NBD β-strands, residues 49–57). Similarly, we included the model of the linker segment, connecting individual NBDs to TMDs, as residues in this region have been shown to play a unique role in coupling ATP hydrolysis to substrate efflux, and the related conformational changes of the transporter [38]. Multiple systems were constructed from the experimental IFS and OFS structures: an apo IFS, a substrate-bound IFS, and a substrate and ATP-Mg^2+^-bound IFS transporter; and an ATP-Mg^2+^-bound OFS, an ADP-bound OFS, and an OFS transporter with no nucleotide bound (see Materials and Methods and SI Table S1 for details). The structures were inserted into a lipid bilayer composed of dimiristoylphosphatidylcholine (DMPC) with 20 % cholesterol (CHOL); the latter has been suggested to play a role in the transport regulation of ABCG2 [33], [39], [40]. We performed classical MD simulations and Normal Mode Analysis (NMA) on all the above systems.

Moreover, we developed an innovative method, kinetically excited targeted MD (ketMD) that traces possible pathways between two terminal structures, that we applied here to simulate conformational transitions between the IFS and the OFS. Our concept relies on the method developed by Costa et al., Molecular Dynamics with excited Normal Modes (MDeNM) [41] designed to enhance protein conformational exploration. In MDeNM, collective motions of the protein described by different combinations of low frequency normal modes are kinetically activated during MD simulations. This enables the coupling of fast and slow degrees of freedom. Recently, we have successfully employed MDeNM to study large functional movements in several biological systems [42], [43] including the gating mechanism of substrate recognition in the sulfotransferase SULT1A1 [44]. As in the case of ABCG2 the target conformation is specified, the excitation vector was chosen to point towards the target structure instead of being a combination of normal modes, similar to targeted MD (tMD) simulations. However, unlike tMD, where the potential energy function is biased and the protein is guided by steering forces at each simulation step, ketMD relies on kinetic excitations. At the first step of each excitation cycle, the velocity components pointing from the instantaneous conformation to the target structure are increased, allowing the crossing of larger energy barriers. This excitation step is followed by a relaxation period where no external perturbation is applied, the system can evolve, and the injected kinetic energy dissipates. After each excitation cycle, the excitation direction vector is updated to point to the target structure from the current conformation. In total, 40 consecutive excitation cycles were performed per system (see Materials and Methods for a detailed description).

### 2.2 Conformational transitions during the ABCG2 transport cycle

The transport cycle of ABCG2 includes two large conformational transitions. Firstly, transition 1, when the IFS transforms into the OFS while the substrate passes from cavity 1 to cavity 2 (from where it is then released to the extracellular space). Secondly, transition 2, when the OFS returns to the initial IFS. The timescale of a complete transport cycle of ABCG2 falls in a range of fraction of seconds or beyond (the initial transport rate of a substrate is 0.1 molecules per ABCG2 dimer per second in the study of Yu et al.) [28], [45], a timeframe that currently cannot be simulated by classical MD. With the help of ketMD, here we present all-atom simulations of transitions 1 and 2 of the membrane-embedded ABCG2. Transition 1 was simulated starting from the IFS with bound E_1_S substrate (an endogenic steroid) and ATP-Mg^2+^, transition 2 from the OFS without bound substrates and in the presence of ATP-Mg^2+^, ADP, or in the absence of bound nucleotides.

#### 2.2.1 Role of the NBDs

**Fig. 2:**
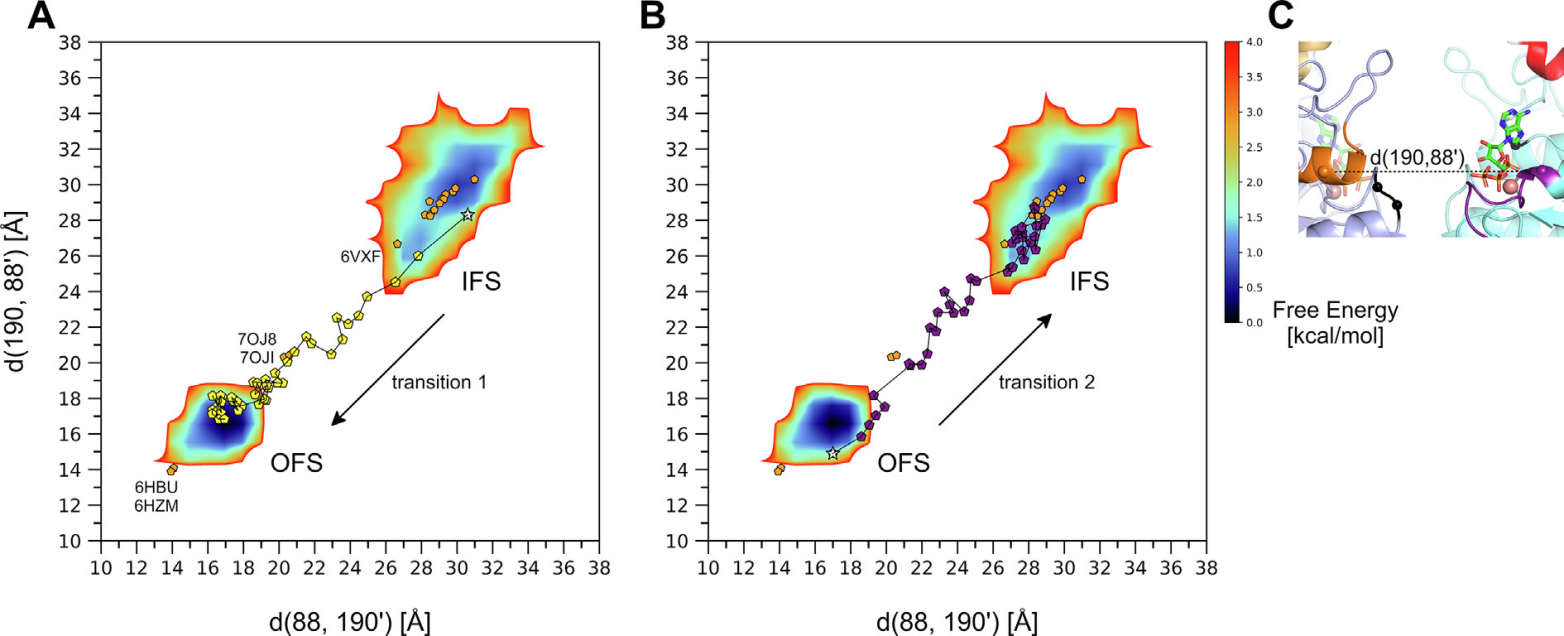
Evolution of the openness at the catalytic ATP-binding site upon (A) transition 1 (yellow pentagons) and (B) transition 2 (purple pentagons) of the ketMD simulations, represented by the distance between the Cα atoms of residues S88 of one monomer and E190 of the other. Free Energy Landscapes (FELs) of the MD-generated conformations starting from the E_1_S- and ATP-Mg^2+^bound IFS and the nucleotide-free OFS are included as references. The initial conformations are indicated as stars, available experimental structures are marked with orange pentagons. (C) The catalytic ATP-binding site and the monitored distance shown in the IFS state. The following experimental structures, which fall in the IFS region, are shown but not labelled in panels A and B: PDB 5NJ3, 6ETI, 6FEQ, 6FFC, 6HCO, 6HIJ, 6VXH, 6VXI, 6VXJ, 7NEQ, 7NEZ, 7NFD, 7OJH. (For interpretation of the references to color in this figure legend, the reader is referred to the web version of this article.)

**Fig. 3:**
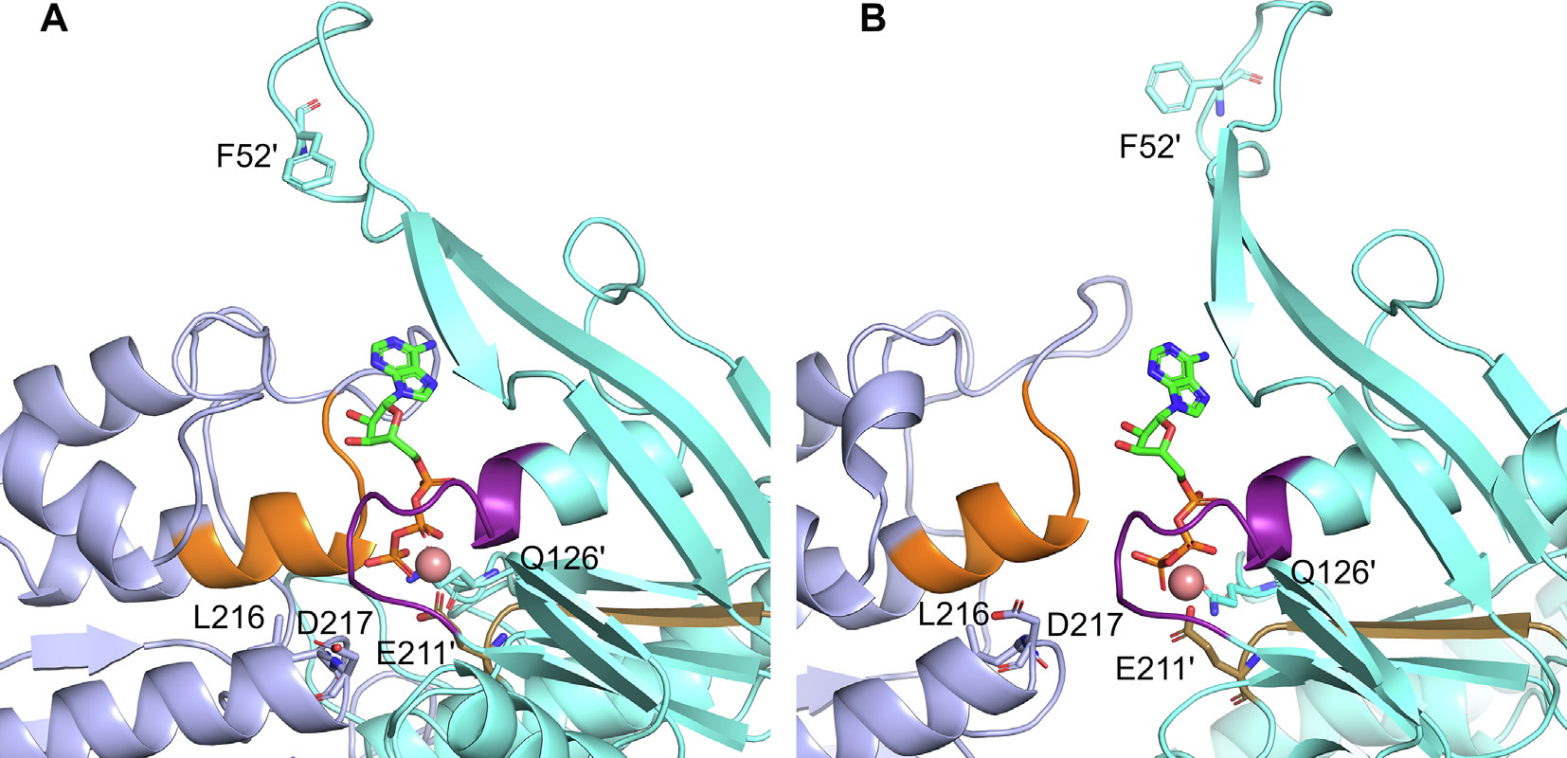
The nucleotide binding site in (A) the OFS cryo-EM structure (PDB 6HBU) and (B) at the end of the ketMD simulation of transition 1. P-loop is highlighted in purple, the signature sequence in orange, and the Walker B motif in tan. The ATP is in licorice, the Mg^2+^ ion in sphere representation. (For interpretation of the references to color in this figure legend, the reader is referred to the web version of this article.)

**Fig. 4:**
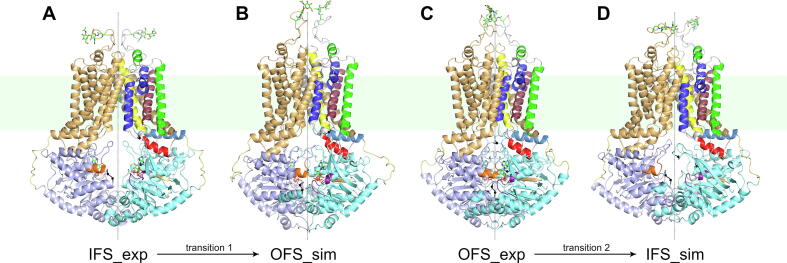
Conformational transitions during the ketMD simulations. (A) The experimental structure (PDB 6HCO) with the modelled missing loops and the added two ATP-Mg^2+^ that was used (after equilibration) as starting structure for the ketMD simulation of transition 1, (B) the final simulated conformation of transition 1, (C) the experimental structure (PDB 6HBU) with the modelled missing loops that was used (after equilibration) as starting structure for the ketMD simulations of transition 2 (either with bound ATP-Mg^2+^, ADP, or no bound nucleotides), (D) the final simulated conformation of transition 1 (in the absence of bound nucleotides). The ATPs are in licorice, the Mg^2+^ ions in sphere representation. The rotational symmetry axis of the homodimer is indicated by dashed lines.

Upon the transition from the IFS to the OFS (transition 1), the two NBDs form a tightly packed dimer. The resulting interface establishes the two catalytic ATP-binding sites between the P-loop (residues 80–88) of one monomer and the signature sequence (‘VSGGERKR’, residues 186-193) of the other [21]. The formation of the two catalytic ATP-binding sites upon the transition of the NBDs can be monitored by the evolution of the distances between the residues at the edges of the P-loop of one monomer and the signature sequence of the other, symmetrically two distances, each corresponding to one of the two ATP-binding sites, namely d(88CA,190′CA) and d(190CA,88′CA) (Fig. 2C). These distances gradually decrease during the ketMD simulation from the initial 30.6 Å and 28.3 Å to less than 17 Å (Fig. 2A). For reference, the distance is around 14 Å in the E211Q mutant ATP-Mg^2+^-bound OFS target structure (PDB 6HBU). This distance averages 15.4 Å across the three 100-ns-long MD runs starting from the wild-type, ATP-Mg^2+^-bound OFS, with values greater than 19 Å present in the trajectories (Fig. 2A,B, Free Energy Landscape (FEL) of the MD generated conformations calculated based on Equation (1) in Materials and Methods) suggesting that during the ketMD simulation of transition 1, the catalytic ATP-binding sites were successfully formed between the P-loop and the signature sequence similarly to what can be observed in the reference OFS MD simulations. The backbone RMSD (root mean square deviation) of the NBD dimer with respect to the target experimental OFS structure was also monitored during the ketMD simulation to follow the closure of the whole NBD region (SI Fig. S1A). The initial RMSD of 7.2 Å gradually decreased to 2 Å during the 40 excitation cycles. As reference, the same RMSD among the classical MD generated OFS conformations is on average 1.8 Å with a standard deviation of 0.23 Å (SI Fig. S1C). Based on these results and visual inspection of the generated conformations (Fig. 3A,B, Fig. 4A,B; Video S1 and Fig. S1A,C of Supplementary Information (SI)), we conclude that a full NBDs transition was successfully achieved together with the catalytic ATP-binding site formation during the ketMD simulation of transition 1.

In the opposite direction, upon the transition from the OFS to the IFS (transition 2), the strong interactions stabilizing the NBD dimer must be broken to obtain the partially separated NBDs, characteristic of the IFS. Some of the strongest interactions exist between the P-loop and d-loop (P81/T82-D217), the P-loop and the signature sequence (T82-R193), and the Q-loop and the signature sequence (D127-R191). The interaction energy between the 2 NBDs is approximately –320 kcal/mol for the MD equilibrated OFS (in the absence of the nucleotides) and –150 kcal/mol for the IFS conformation. Upon the partial separation of the NBDs during the ketMD simulations, we observe a continuous weakening of the interactions (less negative interaction energy), reaching the reference of –150 kcal/mol after the 25th excitation cycle. Simultaneously, the distances d(88CA,190′CA) and d(190CA,88′CA) gradually increase from the initial 17 Å and 14.9 Å to over 28 Å (Fig. 2B), which demonstrates the dissociation of the catalytic ATP-binding sites. The backbone RMSD of the NBD dimer with respect to the target experimental IFS structure (PDB 6HCO) gradually decreased from the initial 7.2 Å to 1.3 Å during the ketMD simulation (SI Fig. S1B). The same RMSD has a mean of 1.7 Å with a standard deviation of 0.27 Å among the classical MD generated IFS conformations (SI Fig. S1D). Visual inspection of the ketMD generated conformations together with the analyses above clearly confirmed that the NBDs got partially separated and a complete NBDs transition occurred (Fig. 4C,D, SI Video S1 and Fig. S1B,D).

To analyze the effect of the presence of ATP, ADP, or the absence of nucleotides on the dissociation of the catalytic ATP-binding sites, we also performed ketMD simulations of transition 2 in the presence of ATP or ADP. During equilibration, the distance between the P-loop and the signature sequence was preserved in the presence of ATP, while it increased slightly in the presence of ADP and without nucleotides. Detaching the NBDs at the ATP-binding site was more easily achieved in the absence of nucleotides, and most difficult in the presence of ATP (SI Fig. S2).

#### 2.2.2 Collapse and recovery of the substrate-binding cavities

**Fig. 5:**
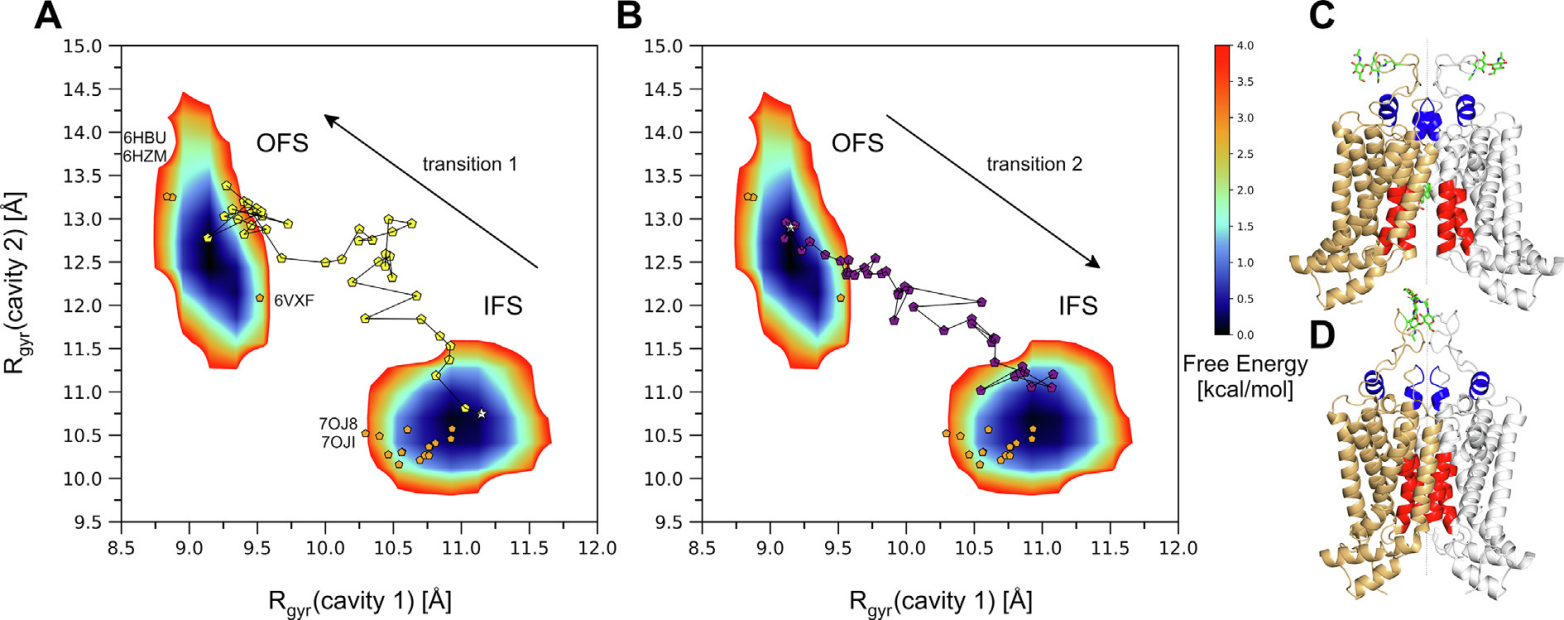
Changes in the substrate-binding cavities represented by the radius of gyration (R_gyr_) of the helical structures bordering the cavities. (A) The collapse of cavity 1 and the opening of cavity 2 during the ketMD simulation of transition 1 denoted by yellow pentagons and (B) the recovery of cavity 1 and the deflation of cavity 2 during the ketMD simulation of transition 2 denoted by purple pentagons. The initial conformations are indicated as stars. Free Energy Landscapes (FELs) of the classical MD generated conformations starting from the E_1_S- and ATP-Mg^2+^-bound IFS and the nucleotide-free OFS are included as reference in panels A and B, available experimental structures are marked with orange pentagons for reference. The structural regions determining the R_gyr_ of cavity 1 (highlighted in red, corresponding to the x-axis of panels A and B) and cavity 2 (highlighted in blue, corresponding to the y-axis of panels A and B) are shown (C) in the IFS experimental structure (PDB 6HCO, open cavity 1 and deflated cavity 2) and (D) in the OFS experimental structure (PDB 6HBU, collapsed cavity 1 and widely open cavity 2). The following experimental structures, which fall in the IFS region, are shown but not labelled in panels A and B: PDB 5NJ3, 6ETI, 6FEQ, 6FFC, 6HCO, 6HIJ, 6VXF, 6VXI, 6VXJ, 7NEQ, 7NEZ, 7NFD, 7OJH. (For interpretation of the references to color in this figure legend, the reader is referred to the web version of this article.)

Experimental data suggest that during the transition from the IFS to the OFS, cavity 1 completely collapses while the previously occluded cavity 2 opens [21], [24], [25], [46]. Once the transporter is reset to its IFS, cavity 1 becomes accessible again. The collapse of cavity 1 occurs as a result of the 2 'coupling helices' (CpH, residues 451–462, corresponding to the C-terminal part of TM2, highlighted in red in Fig. 1) approaching the 2-fold symmetry axis [21]. To assess the changes of the substrate-binding cavities during the conformational transitions, the radius of gyration (R_gyr_) of the helical segments bordering the cavities was calculated. Parts of TM3, TM3’, TM5, and TM5’ (residues 436–446, 436’-446’, 536–547, and 536’-547’) for cavity 1, and the upper part of TM3 and TM3’ (residues 420–425 and 420’-425’) together with the short helical structure within the long loop region connecting TM5 and TM6 (residues 610–617 and 610’-617’) for cavity 2 were included for the R_gyr_ calculations (Fig. 5). The R_gyr_ corresponding to cavity 1 is equal to 10.8 Å in the IFS experimental structure (PDB 6HCO), versus 8.9 Å in the OFS structure (PDB 6HBU) where cavity 1 is completely collapsed. When simulating transition 1 with ketMD starting from the IFS, the R_gyr_ corresponding to cavity 1 was reduced to values under 9.3 Å (Fig. 5A). For reference, the same R_gyr_ has an average of 9.17 Å with a standard deviation of 0.13 Å among the classical MD generated OFS conformations (the heat-maps in Fig. 5A,B correspond to the classical MD simulations). By the end of the ketMD simulation of transition 1, cavity 1 is collapsed and the phenyl rings of residues F439 and F439’, initially stacked against the ring system of E_1_S [21], have moved as close as 3.3 Å from each other, leaving no space for substrates. In the opposite direction starting from the OFS, the R_gyr_ corresponding to cavity 1 increased to as great as 11 Å (Fig. 5B), while cavity 1 became exposed and accessible from the cytosol. The average of the R_gyr_ among the reference classical MD generated IFS conformations is 10.9 Å, the standard deviation is 0.2 Å.

The R_gyr_ corresponding to cavity 2 is equal to 10.3 Å in the IFS, while 13.2 Å in the OFS reference experimental structure (PDB 6HCO and 6HBU respectively). Starting from the IFS, during the ketMD simulation of transition 1 as cavity 2 became more exposed to the extracellular space, it also became more voluminous with R_gyr_ values reaching 13.3 Å (Fig. 5A); while during the simulation of transition 2 starting from the OFS, cavity 2 approached a more deflated state with R_gyr_ values decreasing to around 11 Å. The variations in cavity 2 volume predominantly originate from the rearrangements of the loop regions connecting TM5 and TM6 and the inflating-deflating motions of the cavity are coupled to the conformational transitions between the IFS and the OFS. An additional binding site was proposed by an *in silico* docking study, delimited by TM1, TM2, TM3, and TM4 and formed primarily by residues Q398, S440, S443, R482, and L539′ [47]. Such a site was preserved throughout our ketMD and subsequent classical MD simulations, although E_1_S did not approach it during its translocation in our simulations as it is located more peripherally than cavities 1 and 2, and the leucine gate. That site encompasses residue P480 as well as R482, which was suggested to play an important role in substrate transport [48] and ATP hydrolysis but not in substrate binding [49].

### 2.3 Substrate translocation

In addition to other substrates and inhibitors (SI Table S2), there are currently two E_1_S-bound experimental ABCG2 structures (PDBs 6HCO and 7OJ8). In both cases, the substrate is bound to cavity 1. Experimental structures with a substrate bound to cavity 2, or transient structures along the translocation pathway have not been resolved. With the ketMD run starting from the IFS, it was possible to simulate the translocation of E_1_S from cavity 1 to the extracellular space through the leucine gate and cavity 2. In addition to the excitation applied to the transporter, the substrate motion was also kinetically promoted during our ketMD simulation. The velocity components of its atoms pointing towards the extracellular space, perpendicular to the membrane surface, were repeatedly increased, each time followed by a 5 ps relaxation. Subsequently, we performed 10-ns classical MD simulations starting from the ketMD generated transient conformations along the translocation pathway, to gain insight into the substrate-transporter interactions.

Initially, E_1_S was bound to cavity 1, stabilized mainly by the ‘sandwich-like’ stacking interactions of F439 and F439′. In our ketMD starting conformation, a hydrogen bond formed between N436 and the sulfate group of E_1_S further stabilizes the substrate in cavity 1, although this interaction is non-existent in PDB 7OJ8. The substrate remained bound to cavity 1 until the 7^th^ excitation cycle. Key binding residues may be substrate-dependent, except for F439 which is essential for engaging in the transport, as demonstrated by Gose et al. [50]. The efflux of small molecules investigated in their study was affected by mutation at position F439, but not at N436. However, the latter mutation has been reported to abolish the transport of E_1_S, a bulky compound [21].

**Fig. 6:**
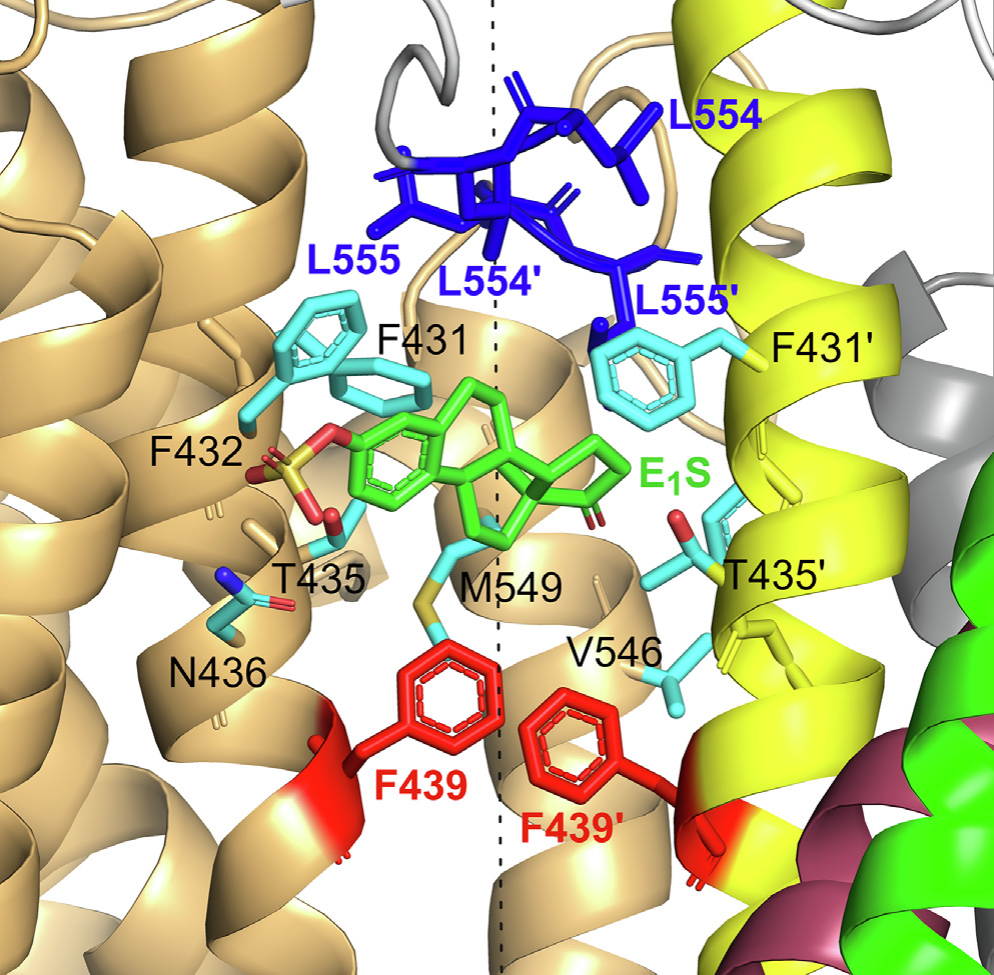
The pocket-like formation observed during the substrate translocation after leaving cavity 1 but before reaching cavity 2, located between the F439 valve (in red) and the leucine gate (in blue). Residues forming strong interactions with the substrate are labelled and are shown in cyan licorice representation. (For interpretation of the references to color in this figure legend, the reader is referred to the web version of this article.)

During the 8^th^ excitation cycle, E_1_S escaped from the ’sandwich-like‘ trap of the two F439 residues and moved towards cavity 2. As soon as the substrate left, F439 and F439′ came into close contact, creating a valve-like construction, similar to what is observed in the OFS cryo-EM structure (PDB 6HBU). Any kind of return movement towards the cytosol is prevented with this valve closed. In our ketMD simulation this was followed by a relatively stable period during which the substrate was trapped between cavities 1 and 2, with movements to cavity 2 still blocked by the closed leucine gate. The stabilizing interactions on the side of this pocket-like formation, located between cavities 1 and 2, involve residues F431, F432, T435, N436, V546, and M549 of the two monomers (Fig. 6 and SI Fig. S3). Interestingly, Krapf et al. have also proposed F431, *F*432, and T435 to interact with quinazolines inhibiting ABCG2 [51]. Our substrate did not move further until the 18^th^ excitation cycle even though the conformational transition continued and the’coupling helices‘ moved closer together. This demonstrates that passing through the leucine gate that necessitates the separation of the leucine residues of the two monomers requires energy. We argue that the conformational transition from the IFS to the OFS alone cannot induce leucine gate opening and substrate passage, as previously suggested for the E211Q mutant by Manolaridis et al. [21]. Our findings are consistent with the observations of Nagy et al., who determined a free energy barrier associated with the substrate passing the leucine gate between 7 and 13 kcal/mol for uric acid, investigated by metadynamics simulations [33].

Once the leucine residues were separated, the substrate was able to slip between them. Here, we identified extensive interactions between E_1_S and the leucine gate, especially L554 and L554′. In addition, strong interactions were formed with Q424, Q424′, F431′, S552′, and F578′. The substrate must first escape the grip of the leucine residues and their surroundings to reach cavity 2. In our simulation, we observed this during the 23^rd^ excitation cycle. In the L554A mutant transporter, possibly reduced attractive interactions may explain its (twofold) higher transport activity than wild-type ABCG2, as reported in the study of Manolaridis et al. [21]. The sulfate group of E_1_S was the last to leave the gate region in our ketMD simulation.

**Fig. 7:**
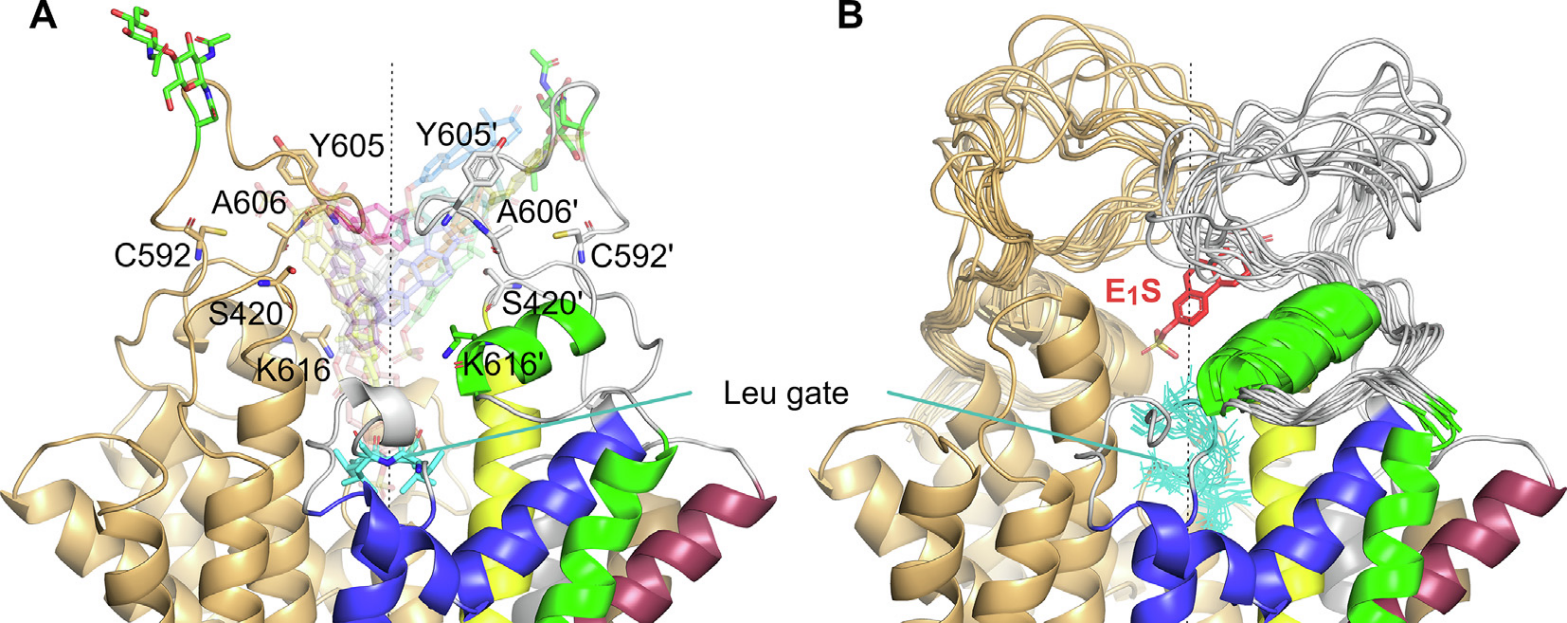
Substrate behavior in cavity 2. (A) Different substrate positions in cavity 2 observed during the classical MD simulations starting from the ketMD-generated transient conformations, from the crossing of the leucine gate to the leaving of the cavity. Residues forming strong interactions with the substrate are labelled and are shown in licorice representation. (B) The fluctuation of the external loop regions corresponding to the substrate positions in panel A.

The substrate behavior in cavity 2 is very different from what can be observed either in cavity 1 or between cavities 1 and 2. Before arriving at cavity 2, the substrate was tightly bound and closely surrounded by transporter residues. In contrast, the substrate was loosely bound here as it explored the cavity volume, making close contacts with residues at its boundary. These contacts involved S420, C592, Y605, and A606 of both monomers and K616 of one of the monomers (Fig. 7 and SI Fig. S4). The substrate’s further kinetic excitation resulted in its complete detachment from the transporter into the extracellular space.

Multiple IFS structures have been resolved with bound nucleotides recently, thus we also performed ketMD simulation of transition 1 starting from the structure PDB 7OJ8 [28] (ATP-bound ABCG2 in the presence of E_1_S in cavity 1). The comparison of the P-loop regions (SI Fig. S5) showed almost no difference between the ATP binding between the PDB 7OJ8 structure and the model we constructed using the IFS structure (PDB 6HCO) with the nucleotide taken from the OFS structure (PDB 6HBU). The NBDs feature a semi-closed dimer in the starting structure (PDB 7OJ8). After some opening during the equilibration, during the ketMD simulation a tightly packed NBD dimer was reached, the catalytic ATP-binding sites were formed (the backbone RMSD of the NBD dimer with respect to the target OFS structure (PDB 6HBU) was decreased from 3.2 Å to 1.7 Å). E_1_S left the grip of the residues F439 and F439′ sooner (after the 3^rd^ excitation cycle) and also its crossing through the leucine gate occurred earlier in the ketMD simulations (after the 12^th^ excitation cycle), compared to the ketMD simulation starting from the more open IFS structure (PDB 6HCO), while cavity 1 collapsed and cavity 2 became more exposed to the extracellular space (the backbone RMSD of the TMD dimer with respect to the target OFS structure was reduced from 3.4 Å to 1.7 Å). The ketMD simulation starting from PDB 7OJ8 further supports the existence of a stable pocket-like formation between cavities 1 and 2 (SI Fig. S6) where E_1_S was trapped for 9 consecutive excitation cycles.

### 2.4 Effect of substrate and nucleotide binding

**Fig. 8:**
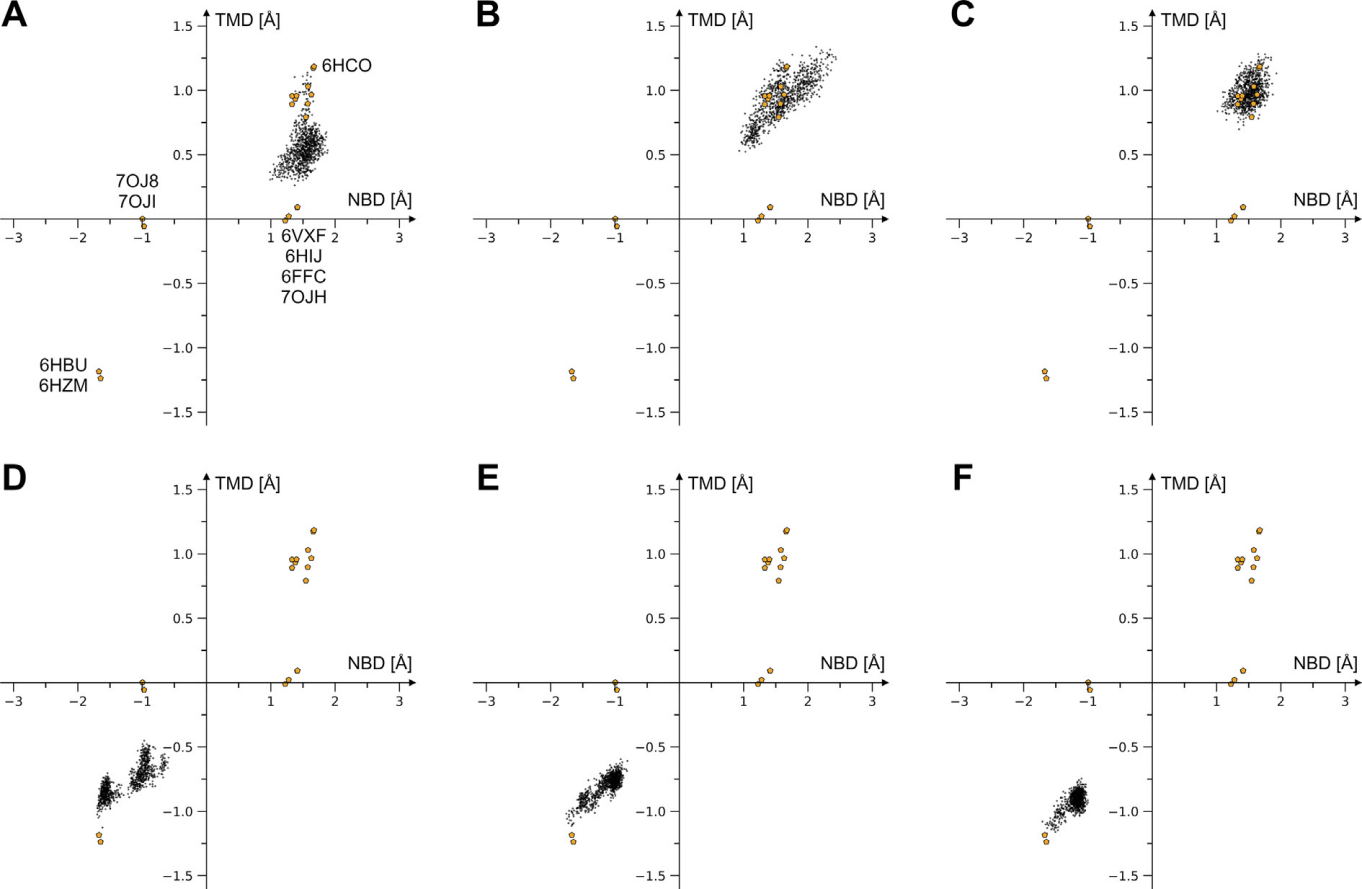
The classical MD generated conformations projected in the subspace of the NBDs’ and TMDs’ difference vectors. The difference vector, after overlapping the IFS (PDB 6HCO) and the OFS (PDB 6HBU) experimental structures, points for each Cα atom from its 3D coordinates in the OFS to its position in the IFS structure. The NBDs and TMDs difference vectors were used for the projection of the conformational differences from the mean experimental structure (of PDB 6HCO and 6HBU) in the case of (A) the apo IFS, (B) the substrate-bound IFS, and (C) the substrate- and ATP-Mg^2+^-bound IFS transporter, (D) the ATP-Mg^2+^-bound OFS, (E) the ADP-bound OFS, and (F) the nucleotide-free OFS ABCG2. Available experimental structures are marked with orange pentagons. The following experimental structures, which fall in the IFS region, are shown but not labelled: PDB 5NJ3, 6ETI, 6FEQ, 6VXI, 6VXJ, 7NEQ, 7NEZ, 7NFD. (For interpretation of the references to color in this figure legend, the reader is referred to the web version of this article.)

We analyzed the different stages of the transport cycle by performing classical MD simulations and NMA. We built IFS systems in their apo-form, with bound E_1_S, and bound E_1_S and ATP-Mg^2+^ together; the OFS systems were constructed with bound ATP-Mg^2+^, ADP, and without nucleotides (for their construction see Materials and Methods and SI Table S1). For all of these systems after equilibration, we first performed a 100-ns classical MD simulation. Fig. 8 shows the MD frames in the subspace of NBDs and TMDs difference vectors. Conformations were first overlapped to the mean-conformation of the IFS and the OFS experimental structures (PDB 6HCO and 6HBU respectively) and were then projected to the NBD and TMD difference vectors, pointing from the OFS to the IFS structure. After overlapping the OFS and the IFS structures, the difference vector points for each Cα atom from its 3D coordinates in the OFS to its position in the IFS structure. The so obtained difference vector of 3 N elements (N is the number of Cα atoms of the system, each having xyz coordinates) was then used to project conformational differences from the mean experimental structure.

We found that the presence of the substrate in cavity 1 is essential to couple the movements between the NBDs and the TMDs. In the absence of a bound substrate and nucleotides (apo-form), the TMDs approach a neutral configuration while the NBDs stay far apart (Fig. 8A). Further analyses revealed that cavity 2 opens while cavity 1 starts collapsing in the absence of a substrate in cavity 1, approaching the state of the apo-closed experimental structure (PDB 6VXF, nucleotide-free apo state), where the arrangement of the TM helices more closely resembles that seen in the outward facing ATP bound state, whereas the lack of NBD dimerization more closely resembles that of the inward facing state [26] (SI Fig. S7A). In contrast, the substrate-bound IFS showed coupled motions between the NBDs and TMDs (Fig. 8B). In the presence of the substrate in cavity 1, we did not observe larger changes in the state of cavities 1 and 2 (SI Fig. S7B,C), the addition of the nucleotides did not induce the onset of a clear transition to the OFS on the simulated time scale (Fig. 8C). We hypothesize that the binding order of substrate and nucleotides related to their physiological concentrations could play a role during the transport cycle, although the time-scale and the character of our simulations do not allow us to draw further conclusions in this regard.

In all the MD simulations starting from the OFS, no conformations moved further away in the direction opposite to the IFS (Fig. 8D,E,F). Moreover, the conformations rapidly drifted in the direction of the IFS, independent of the bound nucleotide. We interpret this as a consequence of removing the E211Q mutation which is present in the only OFS cryo-EM structures available (6HBU and 6HZM). This mutation can generate a more tightly packed NBD dimer-interface than what might exist in the case of the wild-type transporter (see position of E211 in Fig. 3). As a result, it may also generate or stabilize a TMD configuration that is more extremely open to the extracellular space. In all our OFS MD simulations, d(88,190′) exhibited a stable state at around 17 Å (this distance is 13.9 Å in the E211Q mutant OFS cryo-EM structure, 6HBU, SI Fig. S8D,E,F). The R_gyr_ corresponding to cavity 2 slightly decreased (closing of cavity 2) while the R_gyr_ corresponding to cavity 1 slightly increased (opening of cavity 1) or remained unchanged during the nucleotide-bound OFS MD simulations (SI Fig. S7A,B, for cavity 2 the average of ATP-bound OFS is 12.28 Å, ADP-bound OFS is 12.2 Å versus the experimental OFS of 13.2 Å, and for cavity 1 the average of ATP-bound OFS is 9.0 Å, ADP-bound OFS is 9.18 Å versus the experimental OFS of 8.9 Å). This suggests that the most stable states during the nucleotide-bound OFS classical MD simulations were somewhat less extreme than the experimental OFS structure (PDB 6HBU).

Interestingly, in the case of the ATP-Mg^2+^-bound transporter, a steady state was present where one of the ATP-binding site distances d(88,190′) was around 20 Å (SI Fig. S8D). Similar distances exist in the ATP-bound IFS structures in the presence of E_1_S and topotecan (an exogenous substrate), which are 20.3 Å and 20.5 Å, respectively (PDB ID 7OJI and 7OJ8) [28]. It is unclear whether physiologically the ATP-bound OFS is a state with high probability (as the ATPs might be hydrolyzed upon the translocation of the substrate). Our results and the available experimental structures suggest that the sole presence of the ATPs may determine the openness of the NBD dimer.

Furthermore, we argue that contrary to cavity 1, cavity 2 is never fully collapsed, at any stages of the transport cycle even though experiments suggest that it can get occluded [21], [24], [25], [46]. Its volume shows inflating-deflating variations between the IFS and OFS states, predominantly due to the rearrangements of the loop regions connecting TM5 and TM6. However, cavity 2 is more voluminous than cavity 1 even in its deflated state. The restraining region in cavity 2 is the passage between the upper tips of TM3 and TM3′ (residues 420–425). However, this opening shows a high overlap of a large fluctuation between the IFS and the OFS (SI Fig. S9, the average is 7.3 Å and 6.2 Å, the standard deviation is 1.41 Å and 1.39 Å for the IFS and OFS free MD simulations respectively). Moreover, our ketMD and subsequent free MD simulations showed that in the presence of E_1_S at this region, the minimum distance between TM3 and TM3′ can decrease to below 7 Å. This demonstrates that E_1_S, which is a bulky compound could pass through this passage and be present in cavity 2, even in the OFS. It also shows a sufficiently large space for the substrate in cavity 2 throughout the entire transport cycle. This may allow simultaneous substrate binding in cavities 1 and 2, resulting in an accelerated export mechanism.

Prior to our ketMD simulations, we also performed Normal Mode Analysis (NMA) of all the IFS and OFS systems and calculated the fluctuations of the previously discussed distances at 300 K, according to Equation (2) (see Materials and Methods), which we derived from the classical formula of harmonic approximation to the amplitudes of atomic vibrations [52], [53]. We found that the fluctuations of d(88,190′) and d(190,88′) are both significantly damped in the OFS systems compared to the IFS (SI Fig. S10). Visual inspection of the corresponding NMs also revealed that such fluctuations correspond to global transition-like motions in the IFS systems whereas they are local motions of higher frequencies in the OFS systems. Based on the harmonic approximation of NMs, we conclude that it is energetically costly to start transition 2 to return to the initial IFS, which may require the energy released upon ATP hydrolysis, supporting the suggestion for the mechanism by Manolaridis et al. [21].

## 3 Materials and Methods

### 3.1 Transporter structure preparation

All simulations were performed using the human homodimer ABCG2. Cryo-EM structures from the Protein Data Bank were taken as starting coordinates, entry 6HCO [21] (IFS, E_1_S-bound) and 7OJ8 (IFS, ATP- and E_1_S-bound) for the IFSs, and 6HBU [21] (OFS, ATP-Mg^2+^-bound) for the OFSs. The different structural elements of ABCG2 are presented in SI Table S3. The structures PDB 6HCO and 6HBU were solved with the E211Q mutation, which in this study was reverted to the wild type using CHARMM-GUI [54]. The human-specific 5D3 antibody (Fab) molecules were removed from the structure. The missing loop regions in the NBDs (residues 47–60, 302–327, 355–371) were modelled using the DaReUS-Loop web server [55], and the missing C-terminal S655 was built from the internal coordinate table of CHARMM [56]. The first 34 missing *N*-terminal residues were neglected in all our simulations. Disulfide bridges were set between C592 and C608 in each subunit, and between C603 residues linking the two subunits. The PPM web server [57] was used to determine the orientation of the transporter within the membrane. The pKa values of the protein titratable groups were calculated with PROPKA [58], and protonation states were assigned at pH 7.0 outside, and pH 4.0 inside the membrane. The parameters of the substrate E_1_S molecule were determined using the CHARMM General Force Field (CGenFF) 2.5 [59].

Multiple systems were constructed starting from the experimental IFS and OFS structures. Using PDB 6HCO an apo IFS (by removing E_1_S from cavity 1), a single substrate-bound IFS, and a substrate- and ATP-Mg^2+^-bound IFS transporter (by taking the ATP-Mg^2+^ positions after overlapping the backbone residues 80–94 of 6HCO on 6HBU (RMSD of 0.6 Å), SI Fig. S11). An additional ATP-Mg^2+^-bound IFS transporter was constructed using PDB 7OJ8. Furthermore, using the structure PDB 6HBU an ATP-Mg^2+^-bound OFS, an ADP-bound OFS (by cleaving away the γ-phosphates of the ATPs *in silico* and removing the Mg^2+^ ions), and an OFS transporter with no nucleotides bound (by removing both the ATPs and the Mg^2+^ ions, see SI Table S1 for details).

The PPM-oriented structures were inserted into a lipid bilayer composed of dimiristoylphosphatidylcholine (DMPC) with 20 % cholesterol (CHOL) following the work of Ferreira et al. [39] and the TIP3-solvated systems were generated by CHARMM-GUI. The NaCl concentration was set to 0.15 M.

Each system was energy minimized by alternating 250 steps Steepest Descent (SD) and 250 steps of Adopted Basis Newton-Raphson (ABNR) minimization, 10 times each. This was followed by 10,000 Conjugate Gradient (CONJ) steps. The minimization steps were performed with CHARMM [56] using the all-atom additive CHARMM C36m [60] force field (FF), with harmonic constraints applied to the backbone (10 kcal/mol/Å^2^) and the side chain (5 kcal/mol/Å^2^) heavy atoms.

The systems were then equilibrated at 300 K with progressively decreasing harmonic restraining force constants (every 100 ps) by adopting the values 10, 5, 2.5, 1, 0.5, 0.1 kcal/mol/Å^2^ for the backbone heavy atoms and 5, 2.5, 1.25, 0.5, 0.25, 0.05 kcal/mol/Å^2^ for the side chain heavy atoms in an NVT ensemble. The pressure was set to 1 atm and the integration time step to 1 fs. Finally, a 5 ns NPT equilibration run was performed at 300 K, 1 atm, with an integration time step of 2 fs. Equilibration runs were performed with NAMD [61] using the C36m FF. Langevin dynamics was used for constant temperature control with a damping coefficient of 1 ps^−1^. Constant pressure was achieved using the Nose-Hoover method with a piston oscillation period of 50 fs and a piston oscillation decay time of 25 fs. For energy calculations, the dielectric constant was set to 1. The Particle Mesh Ewald (PME) method was used to calculate electrostatic interactions with a grid spacing of 1 Å or less having the order of 6. The real-space summation was truncated at 12.0 Å, and the width of Gaussian distribution was set to 0.34 Å^−1^. Van der Waals interactions were reduced to zero by ‘switch’ truncation operating between 10.0 and 12.0 Å.

### 3.2 Molecular dynamics simulations

Three, 100-ns-long classical Molecular Dynamics (MD) simulations with different initial velocity distributions were carried out on the systems that were also used in our ketMD simulations (the substrate- and ATP-Mg^2+^-bound IFS based on PDB 6HCO and the OFS with no bound nucleotide based on 6HBU), using the same initial conformations as ketMD in order to compare the conformational space exploration of MD to the ketMD simulations. A single 100-ns-long MD simulation was carried out for the other four IFS and OFS systems listed previously. NAMD was used for all of these runs with the C36m FF. The integration time step was 2 fs, and the coordinates were saved every 10 ps. The same parameters were used as for the 5 ns NPT equilibration runs described above. Further 10 ns classical MD simulations were carried out starting from the transient conformations along transitions 1 and 2, generated by the ketMD simulations to identify transporter-substrate interactions along the translocation pathway. The systems with the transient conformations were first de-excited by releasing the excess kinetic energy introduced along the excitation target direction for 10 ps before starting the classical MD simulations. This was achieved by applying a harmonic restraint potential along the target direction, allowing the fast dissipation of the excitation energy. The applied harmonic force constant was 1000 kcal/mol/Å^2^.

### 3.3 Normal Mode Analysis

Normal Mode Analysis (NMA) of each system was performed using the same C36m FF, starting from the equilibrated conformations. The lipid and solvent molecules were first removed. The potential energy of the transporter with the bound ligands was then energy minimized using the SD method with decreasing harmonic restraining potentials applied to the heavy atoms. The harmonic restraining force constants were decreased every 500 steps adopting the values 10, 1, 0.1, and 0 kcal/mol/Å^2^. ABNR minimization followed until an RMS energy gradient of 10^-6^ kcal/mol/Å was reached. The normal modes of the energy minimized structures were calculated using the iterative Mixed-Basis Diagonalization (DIMB) routine [62], [63] of the VIBRAN module in CHARMM.

### 3.4 Kinetically excited targeted MD

We implemented a method, kinetically excited targeted MD (ketMD), to simulate the conformational transitions between the IFS and the OFS. Our concept relies on the MDeNM method [41], designed to enhance the conformational exploration of proteins. Similar to MDeNM, ketMD is based on kinetic excitations. In each excitation cycle, the velocity components pointing from the instantaneous conformation to the target are increased at the first step of the MD simulation. Then, the injected kinetic energy dissipates during a relaxation period while no external perturbation is introduced, and the system progresses. The kinetic excitation corresponded to an overall temperature rise of 2 K in the systems (as was suggested by Kaynak et al. [64] for MDeNM). As the excitation kinetic energy dissipates rapidly (in less than 1 ps [41], [65]), 40 consecutive excitation cycles were performed, each containing a 5 ps relaxation MD simulation. Thus, the total ketMD simulation time was 40 × 5 ps = 200 ps per system. We performed ketMD simulations with excitation applied also to the substrate, starting from the substrate-bound IFS. The velocity components of the substrate, perpendicular to the membrane surface pointing to the extracellular space, were also increased at the first step of the MD simulations in each excitation cycle, corresponding to an additional 0.5 K temperature rise of the given system.

### 3.5 Free energy Landscape (FEL) calculations

FELs of the MD-generated conformations were calculated within the subspace of d(88,190′) vs d(190,88′) and the R_gyr_ corresponding to cavities 1 and 2. The most populated state was used as a reference for calculating free energy differences. The free energy difference (*ΔG_α_*) of a given state *α* was determined by considering the probability of the occurrence of the states *P(q_α_)* and *P_max_(q)* given by the equation:

(1) $$\Delta G_{\alpha }={-k}_{B}Tln\left[\frac{P(q_{\alpha })}{P_{\max}\left(q\right)}\right]$$

where *k_B_* is the Boltzmann constant, *T* is the temperature of the simulation, *P(q_α_)* is an estimate of the probability density function obtained from the bi-dimensional histogram of the conformational distribution, and *P_max_(q)* is the probability of the most populated state. The free energy differences should be considered here as entropic quantities reflecting the populations in terms of energy.

### 3.6 Distance RMSF in NMs

The harmonic approximation to the amplitudes of inter-atomic distance vibration contributions by the different NMs at a given temperature was calculated by evaluating the equation:

(2) $$\langle \Delta d_{p,q,i}^{2}\rangle =\frac{k_{B}T}{\omega_{i}^{2}}{\|\frac{e_{q,i}}{\sqrt{m_{q}}}-\frac{e_{p,i}}{\sqrt{m_{p}}}\|}^{2}$$

where ***d****_(p,q,i)_* is the instantaneous distance vector between atom *p* and *q* in the *i*^th^ NM, *k_B_* the Boltzmann constant, *T* the absolute temperature of the system, *ω_i_* the frequency of the *i*^th^ NM, ***e****_(p,i)_* and ***e****_(_*_q,_*_i)_* the mass-weighted displacement vectors of atom *p* and *q* in the *i*^th^ NM, and *m_p_* and *m_q_* the mass of atoms *p* and *q*, respectively.

### 3.7 Interaction energies

The interaction energy (E_int_) between two groups of atoms was calculated as a sum of pairwise non-bonded electrostatic and van der Waals energies. For the energy calculations CHARMM was used with a distance dielectric constant of 2. The interactions were calculated by considering the atoms of the substrate and a given transporter residue. The energy values reported are statistical averages of the given E_int_ calculated among the conformations retrieved from the free MD simulations.

## 4 Conclusions

In this study, we developed and employed an innovative enhanced MD simulation approach, termed ketMD (kinetically excited targeted Molecular Dynamics), which uses kinetic excitation to promote protein movements corresponding to large conformational changes towards a specified target structure, without biasing the potential energy function. With the help of ketMD, we successfully simulated the conformational transitions of the ABCG2 transport cycle, and revealed the complex molecular mechanism of the physiological E_1_S substrate translocation. We observed a valve-like function of residues that initially engage in stacking interactions against the substrate in cavity 1 (F439 and F439′). We found that they prevent backwards movements of the substrate towards the cytosol once it escapes their grasp and moves towards the leucine gate. We also identified a pocket-like construction between this valve and the leucine gate, where the substrate is stabilized before it moves to cavity 2.

Furthermore, using MD simulations and NMA of the different transport stages, we have shown that the presence of the substrate in cavity 1 is essential to couple the movements between the NBDs and the TMDs. Additionally, we observed that cavity 2 was never completely collapsed, at any stages of the transport cycle. Therefore, we hypothesize that simultaneous substrate binding in cavities 1 and 2 may occur, which could result in an accelerated export mechanism.

Finally, the harmonic approximation of the ABCG2 dynamics by NMA revealed that low frequency global transition-like motions exist in the IFS but were absent in our calculations for the OFS transporter, where partial transition-like movements are present but are more localized and of higher frequencies. Accordingly, our results further support previous assumptions that transition 2, starting from the OFS transporter, is energetically costly and ABCG2 requires the energy released upon ATP hydrolysis to return to its initial IFS.

Our observations shed new light on the complex molecular mechanism of the ABCG2 transport, and the results highlighted the utility of including enhanced *in silico* sampling techniques, such as ketMD, in transporter studies. In the future, the provided transition pathways can help to identify novel ABCG2 substrates and inhibitors, and probe new drug candidates for MDR and DDI.

## Funding

B.D. has received funding from Université Paris Cité (the Idex project).

## Declaration of Competing Interest

The authors declare that they have no known competing financial interests or personal relationships that could have appeared to influence the work reported in this paper.
