# Supplementary material for: ABCG2/BCRP transport mechanism revealed through kinetically excited targeted molecular dynamics simulations
Source: Comput Struct Biotechnol J. 2022 Jul 29;20:4195–205. doi: 10.1016/j.csbj.2022.07.035 (PMC9389183; doi:10.1016/j.csbj.2022.07.035)
Supplement: Supplementary data 1 [file mmc1.pdf]

Supporting Information

ABCG2/BCRP transport mechanism revealed through kinetically excited targeted molecular dynamics simulations

B. Dudas[1,2], X. Decleves[3,4], S. Cisternino[3,5], D. Perahia[2,*], M. A. Miteva[1,*]

[1]Inserm U1268 MCTR, CiTCoM UMR 8038 CNRS - Université Paris Cité, Paris, France
[2]Laboratoire de biologie et pharmacologie appliquée, Ecole Normale Supérieure Paris-Saclay, Gif-sur-Yvette, France
[3]Inserm UMRS 1144, Optimisation Thérapeutique en Neuropsychopharmacologie - Université Paris Cité, Paris, France
[4]Biologie du Médicament et Toxicologie, Assistance Publique Hôpitaux de Paris, AP-HP, Hôpital Universitaire Cochin, Paris, France
[4]Service Pharmacie, Assistance Publique Hôpitaux de Paris, AP-HP, Hôpital Universitaire Necker-Enfants Malades, Paris, France

[*]Corresponding authors (maria.mitev@inserm.fr, david.perahia@ens-paris-saclay.fr)

**State**	**Name of System**	**Ligands**			**Initial PDB ID**	**Details on System Assembly**
		**E_1S**	**ATP-Mg^{2+}**	**ADP**		
IFS	apo IFS				6HCO	E_1S removed from cavity 1
	E_1S bound IFS	✓				
	E_1S & ATP-Mg^{2+} bound IFS *	✓	✓			ATP-Mg^{2+} positions taken from 6HBU after overlapping on residues 80-94
OFS	ATP-Mg^{2+} bound OFS		✓		6HBU	
	ADP bound OFS			✓		ATP γ-phosphates cleaved, Mg^{2+} ions removed
	nucleotide-free OFS					ATPs and Mg^{2+} ions removed

Table S1: Details on the different systems used in the MD simulations and NMA.
(*) An E_1S and ATP-Mg^{2+}-bound IFS was also constructed using the structure PDB 7OJ8.

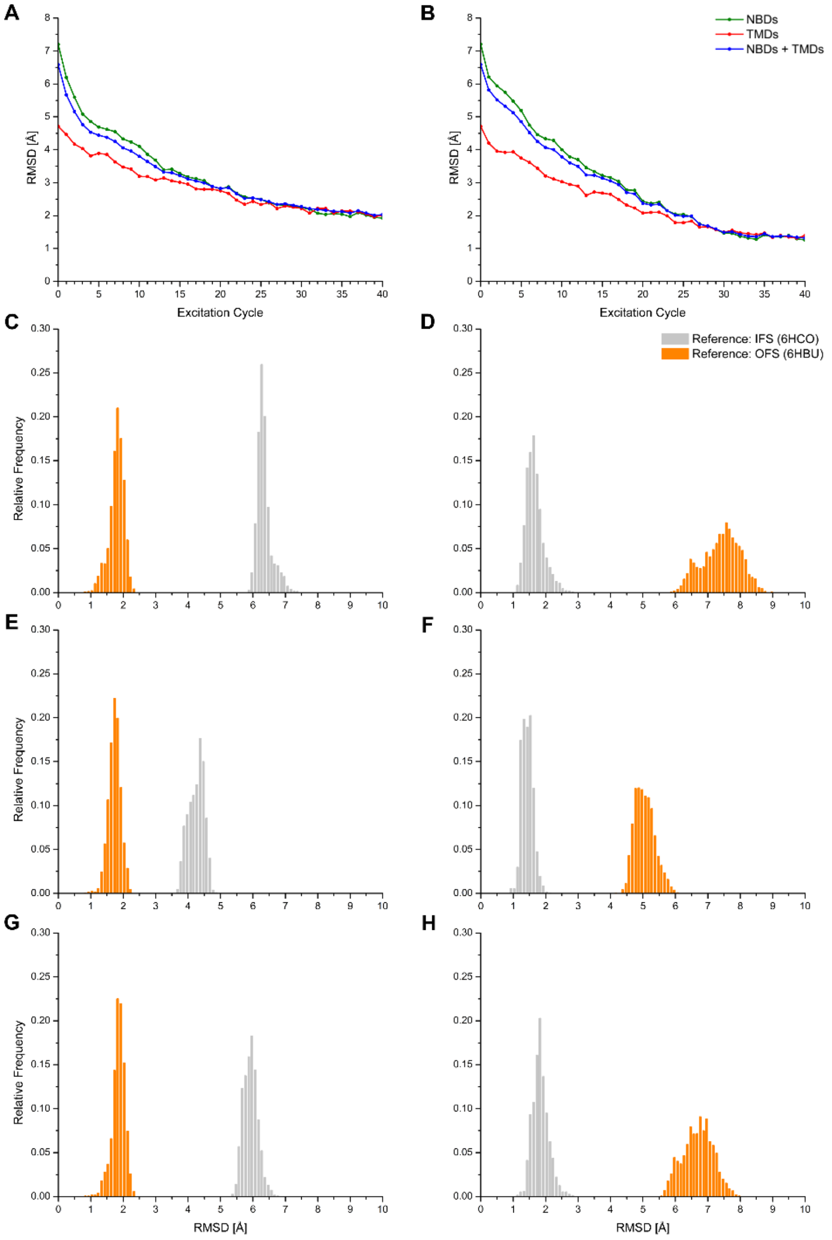

Figure S1: Root Mean Square Deviation (RMSD) from experimental reference structures. Evolution of the backbone RMSD during the ketMD simulation **(A)** of transition 1 and **(B)** of transition 2 with respect to their corresponding experimental target structures (PDB 6HBU (OFS) for transition 1 and PDB 6HCO (IFS) for transition 2); RMSD calculated on the NBD dimer is in olive, on the TMD dimer in red, and on the whole transporter in blue. RMSD distribution of the three 100-ns-long classical MD generated conformations, calculated on the NBD dimer for **(C)** the OFS and **(D)** the IFS, calculated on the TMD dimer for the **(E)** OFS and **(F)** the IFS, and calculated on the whole transporter for the **(G)** OFS and the **(H)** IFS MD conformations. RMSD with respect to PDB 6HCO (IFS) is in light gray, with respect to PDB 6HBU (OFS) in orange.

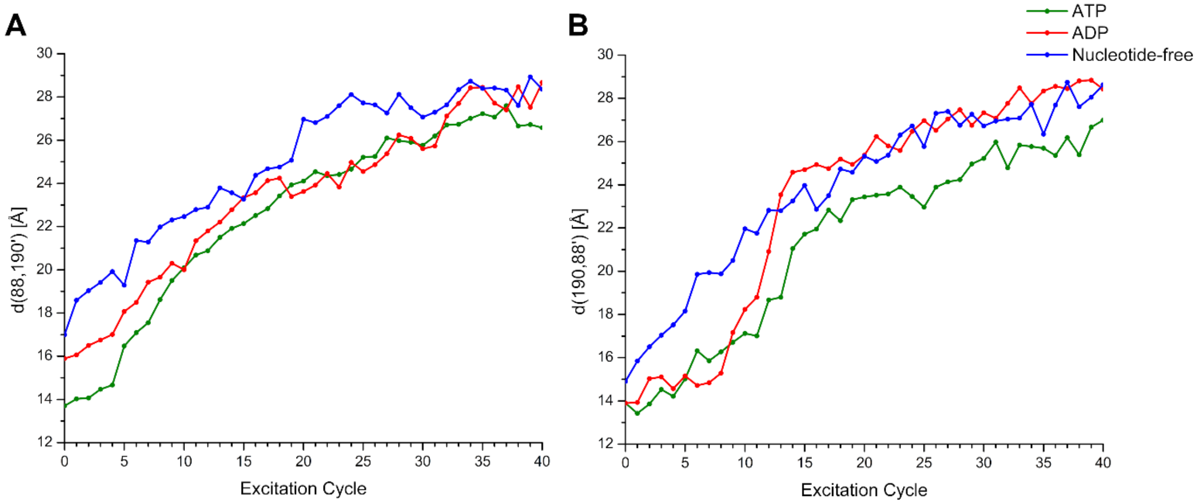

Figure S2: The evolution of the distances corresponding to the two catalytic ATP-binding sites, symmetrically between the P-loop of one monomer and the signature sequence of the other, represented by the distance **(A)** between the Cα atoms of residues S88 and E190' and **(B)** between the Cα atoms of residues E190 and S88' during the ketMD simulations of transition 2. The ketMD simulation in the presence of ATP-Mg^{2+} is shown in olive, in the presence of ADP in red, and in the absence of bound nucleotides in blue. The protein initial conformation of all three cases correspond to the experimental structure PDB 6HBU.

PDB	Ligand*	Nucleotide	State	Reference
5NJ3	-	-	IFS	[1]
6HIJ	MZ29 (*I*)	-	IFS	[2]
6FFC	MZ29 (*I*)	-	IFS	
6FEQ	FKo143 (*I*)	-	IFS	
6ETI	MZ29 (*I*)	-	IFS	
6HCO	estrone 3-sulfate (*S*)	-	IFS	[3]
6HBU	-	ATP-Mg^{2+}	OFS	
6HZM	-	ATP-Mg^{2+}	OFS	
6VXF	-	-	IFS/OFS**	[4]
6VXJ	SN38 (*S*)	-	IFS	
6VXI	mitoxantrone (*S*)	-	IFS	
6VXH	imatinib (*S*)	-	IFS	
7NFD	mitoxantrone (*S*)	-	IFS	[5]
7NEZ	topotecan (*S*)	-	IFS	
7NEQ	tariquidar (*S*)	-	IFS	
7OJI	topotecan (*S*)	ATP	IFS/OFS***	[6]
7OJH	topotecan (*S*)	ATP	IFS	
7OJ8	estrone 3-sulfate (*S*)	ATP	IFS/OFS***	

Table S2: Available ABCG2 experimental structures.
(*) *S* = substrate, *I* = inhibitor
(**) 'apo-closed state', the arrangement of TM helices more closely resembles that seen in the outward facing ATP bound state, whereas the lack of NBD dimerization more closely resembles that of the inward facing state [4]
(***) 'turnover-2 state', semi-closed NBDs and an almost fully occluded substrate cavity [6]

1. Taylor, N.M.I., et al. (2017) *Structure of the human multidrug transporter ABCG2.* Nature. 546:504-509.
2. Jackson, S.M., et al. (2018) *Structural basis of small-molecule inhibition of human multidrug transporter ABCG2.* Nat Struct Mol Biol. 25:333-340.
3. Manolaridis, I., et al. (2018) *Cryo-EM structures of a human ABCG2 mutant trapped in ATP-bound and substrate-bound states.* Nature. 563:426-430.
4. Orlando, B.J. and M. Liao (2020) *ABCG2 transports anticancer drugs via a closed-to-open switch.* Nat Commun. 11:2264.
5. Kowal, J., et al. (2021) *Structural Basis of Drug Recognition by the Multidrug Transporter ABCG2.* J Mol Biol. 433:166980.
6. Yu, Q., et al. (2021) *Structures of ABCG2 under turnover conditions reveal a key step in the drug transport mechanism.* Nat Commun. 12:4376.

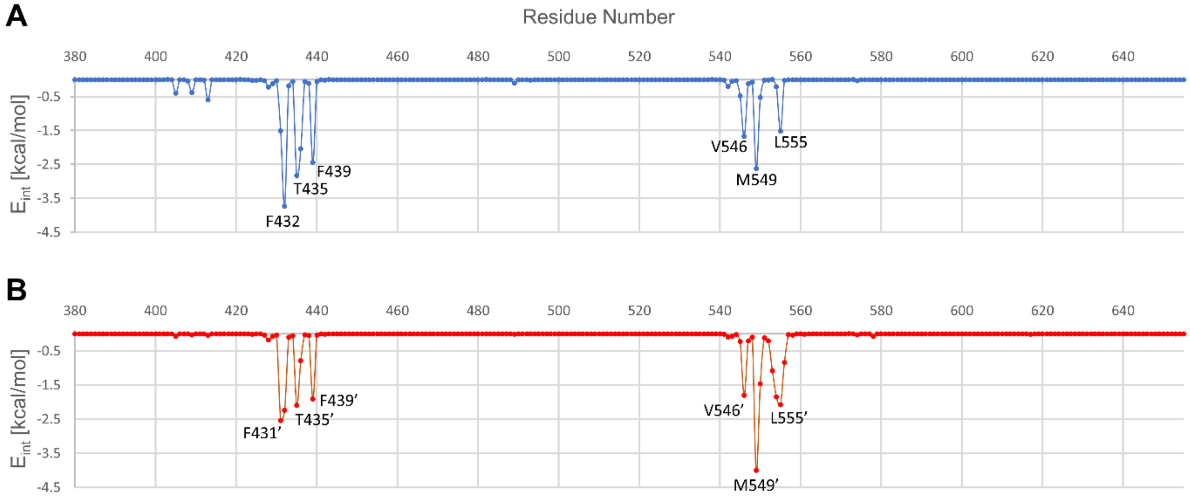

Figure S3: Interaction Energy between the residues of the transporter and the E_1S substrate when trapped in the pocket-like formation between the F439 valve and the leucine plug, based on the classical MD simulations starting from the ketMD generated transient conformations for (**A**) one monomer and (**B**) the other.

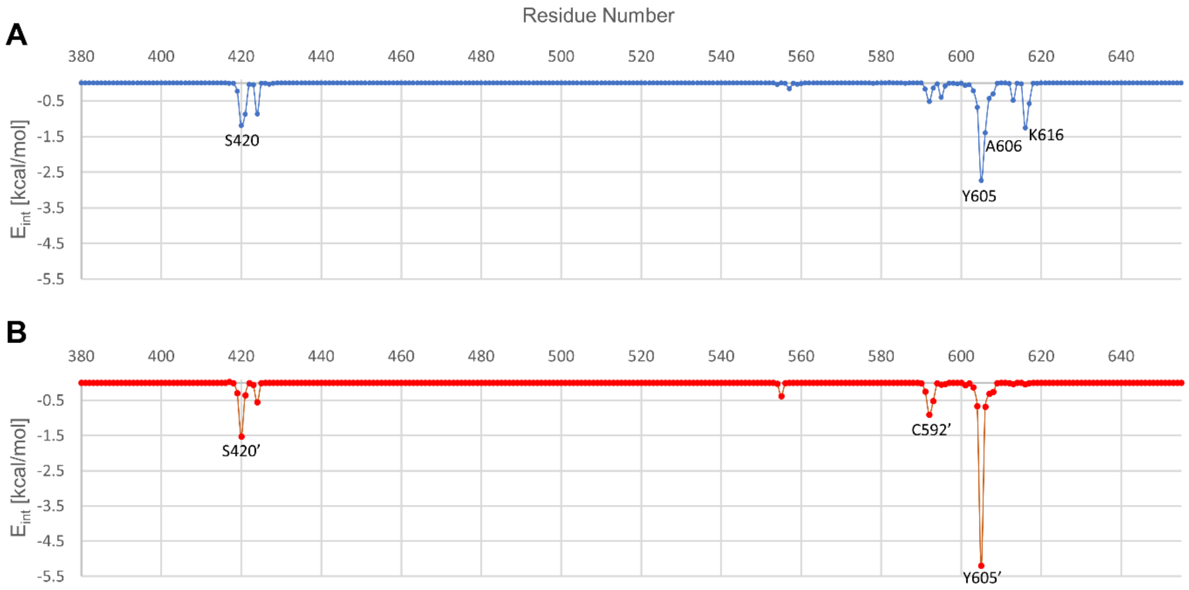

Figure S4: Interaction Energy between the residues of the transporter and the E_1S substrate in cavity 2, based on the classical MD simulations starting from the ketMD generated transient conformations for (**A**) one monomer and (**B**) the other.

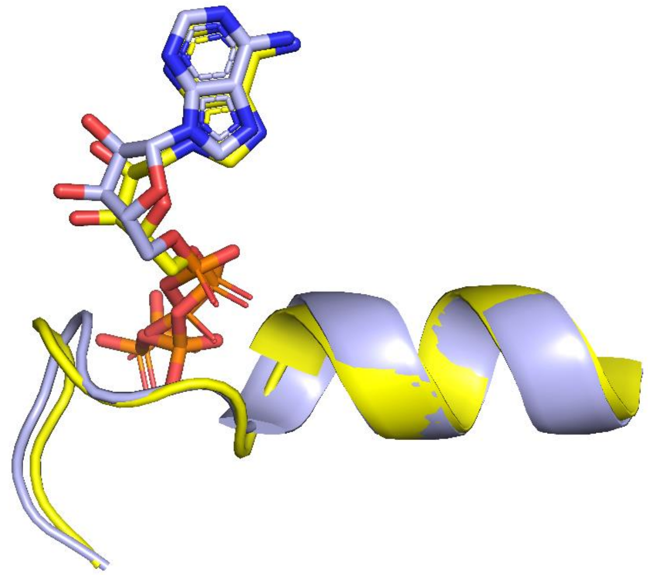

Figure S5: The superposition of residues 80-94 of the PDB 7OJ8 structure (in light blue) with bound ATP and the model (in yellow) that was constructed using the IFS structure (PDB 6HCO) with the nucleotide from the OFS structure (PDB 6HBU). The ATPs are in licorice representation.

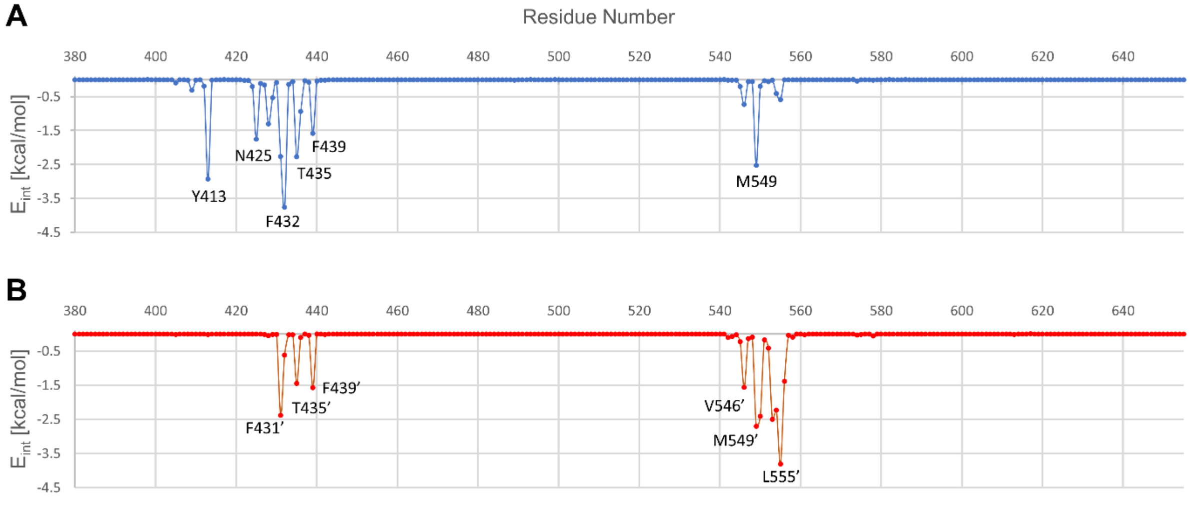

Figure S6: Interaction Energy between the residues of the transporter and the E_1S substrate when trapped in the pocket-like formation between the F439 valve and the leucine plug for (**A**) one monomer and (**B**) the other. The interaction energies are based on the classical MD simulations that were performed starting from the transient conformations of the ketMD simulation using the initial structure PDB 7OJ8.

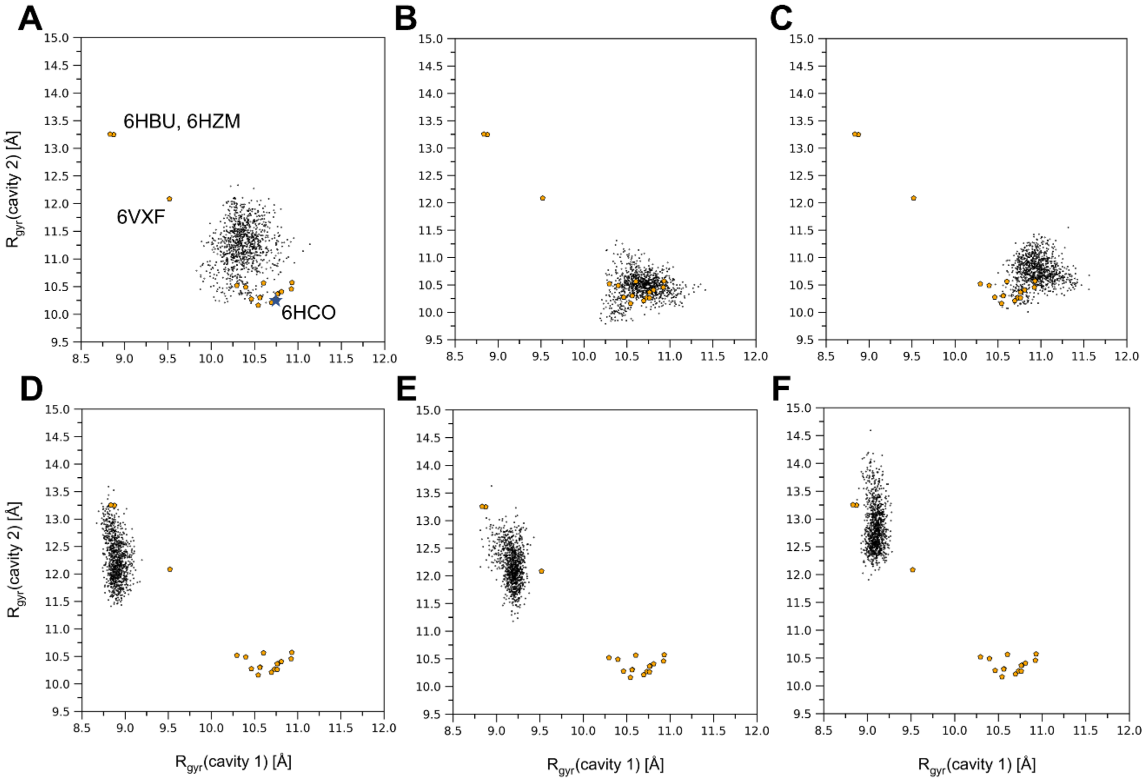

Figure S7: Changes in the substrate-binding cavities represented by the radius of gyration (R_{gyr}) of the helical structures bordering the cavities during classical MD simulations. Conformations of (**A**) the apo IFS, (**B**) the substrate-bound IFS, and (**C**) the substrate- and ATP-Mg^{2+}-bound IFS transporter, and (**D**) the ATP-Mg^{2+}-bound OFS, (**E**) the ADP-bound OFS, and (**F**) the nucleotide-free OFS simulations. Available experimental structures are marked with orange pentagons.

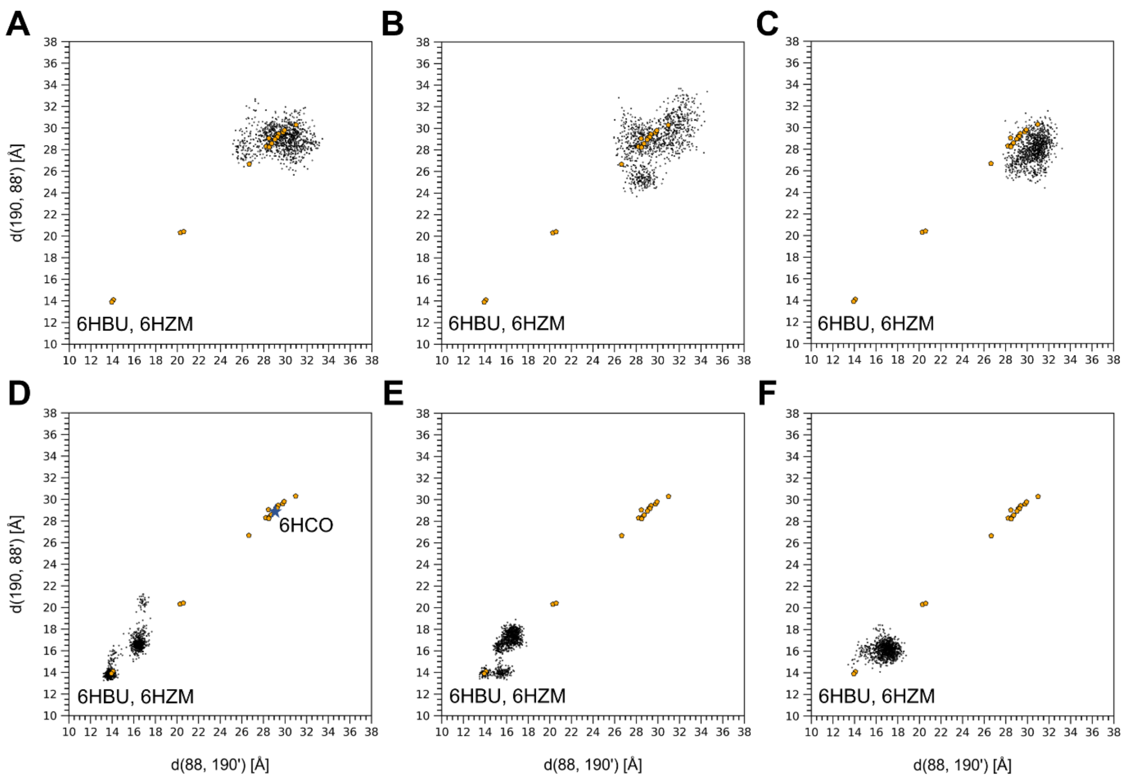

Figure S8: The openness at the catalytic ATP-binding site of the MD generated conformations, represented by the distance between the CA atoms of residues S88 of one monomer and E190 of the other. Simulations on (**A**) the apo IFS, (**B**) the substrate bound IFS, and (**C**) the substrate and ATP-Mg^{2+} bound IFS transporter, (**D**) the ATP-Mg^{2+} bound OFS, (**E**) the ADP bound OFS, and (**F**) the nucleotide-free OFS ABCG2. Available experimental structures are marked with orange pentagons.

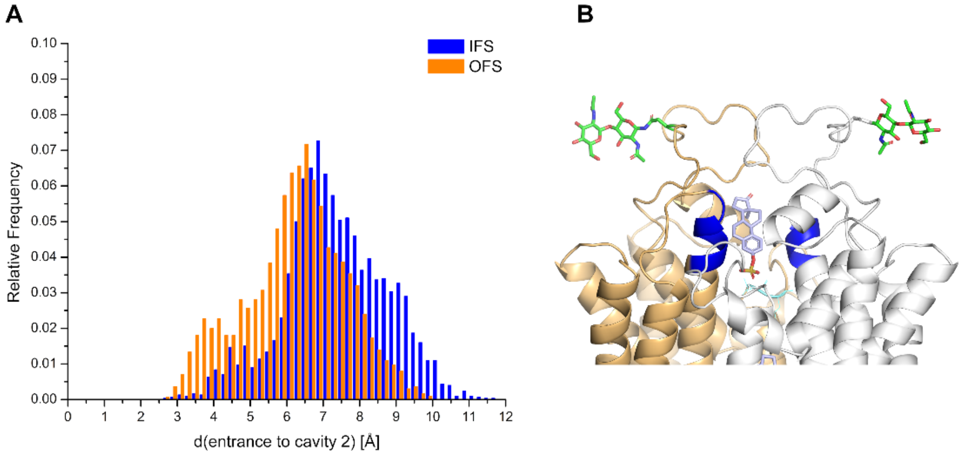

Figure S9: The tightest part of cavity 2 after passing through the leucine gate. **(A)** The population distribution of the minimum distance between the upper tip of TM3 (residues 420-425) and TM3' (residues 420'-425') heavy atoms. The distribution of the IFS is shown in blue, and the OFS classical MD simulations in orange. **(B)** The regions at the upper tips of TM3 and TM3' which were used to calculate the minimum distance in panel A.

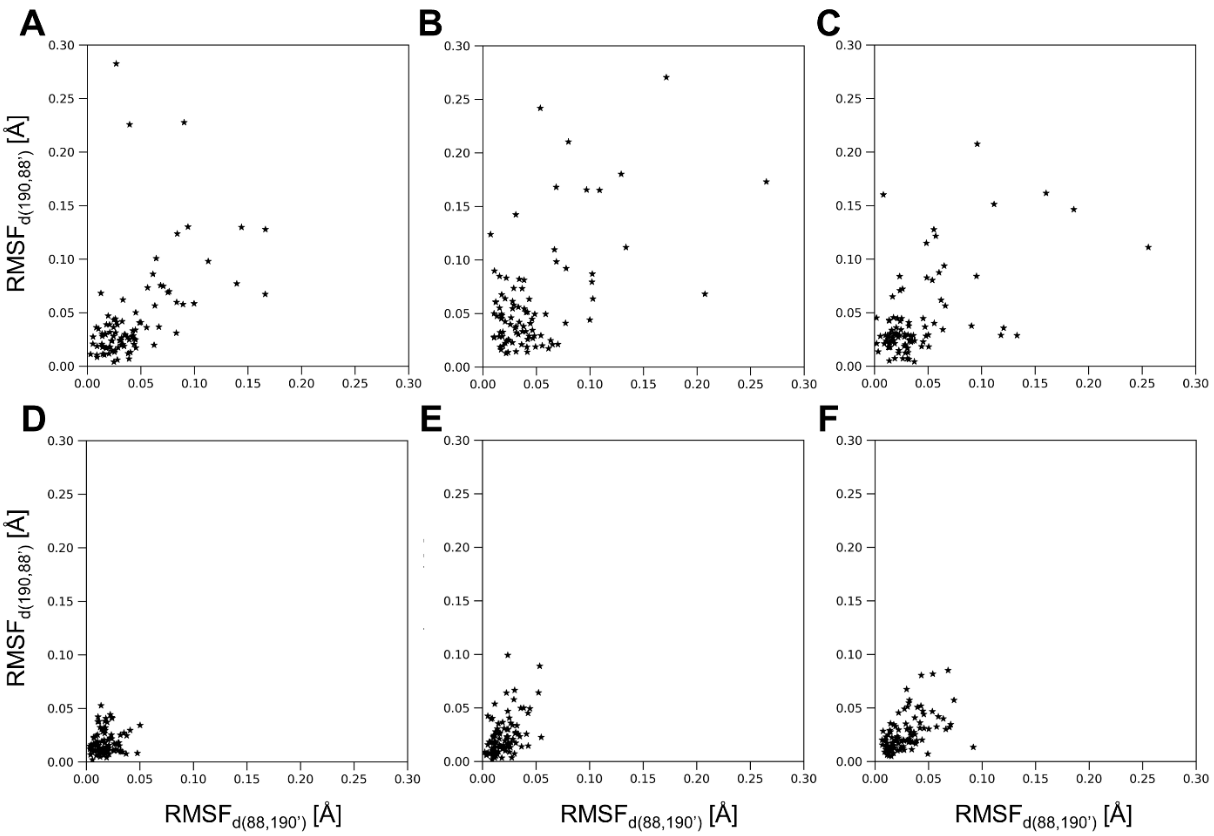

Figure S10: Amplitudes of NM contributions to the fluctuations of the distances between the CA atoms of residues S88 of one monomer and E190 of the other at 300 K, in the case of (**A**) the apo IFS, (**B**) the substrate-bound IFS, and (**C**) the substrate- and ATP-Mg^{2+}-bound IFS transporter, (**D**) the ATP-Mg^{2+}-bound OFS, (**E**) the ADP-bound OFS, and (**F**) the nucleotide-free OFS ABCG2.

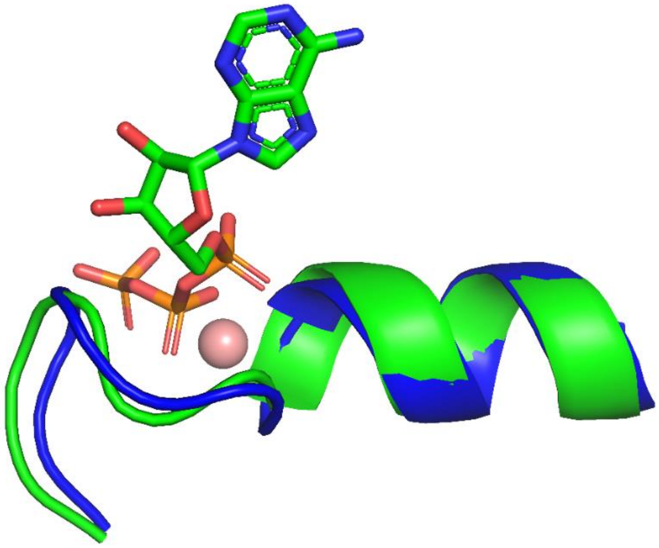

Figure S11: The superposition of residues 80-94 of the IFS (6HCO, blue) and the OFS structure (6HBU, green). The ATP is in licorice, the Mg^{2+} ion in sphere representation.

Domain	Region	Residue(s)
NBD	A-loop	F52
	P-loop (Walker A)	80-88
	Q-loop	Q126
	Signature sequence	186-193
	Walker B	206-211
	D-loop	L216, D217
	H-loop	H243
TMD	Elbow helix (TM1a)	373-391
	TM1b	393-413
	TM2	421-448
	CpH	451-461
	TM3	466-496
	TM4	503-528
	TM5a	535-552
	TM5b	565-571
	TM5c	573-585
	TM6a	610-617
	TM6b	623-650

Table S3: Structural elements of ABCG2.
